# Brugada syndrome genetics is associated with phenotype severity

**DOI:** 10.1093/eurheartj/ehaa942

**Published:** 2020-11-21

**Authors:** Giuseppe Ciconte, Michelle M Monasky, Vincenzo Santinelli, Emanuele Micaglio, Gabriele Vicedomini, Luigi Anastasia, Gabriele Negro, Valeria Borrelli, Luigi Giannelli, Francesca Santini, Carlo de Innocentiis, Roberto Rondine, Emanuela T Locati, Andrea Bernardini, Beniamino C Mazza, Valerio Mecarocci, Žarko Ćalović, Andrea Ghiroldi, Sara D’Imperio, Sara Benedetti, Chiara Di Resta, Ilaria Rivolta, Giorgio Casari, Enrico Petretto, Carlo Pappone

**Affiliations:** Arrhythmia and Electrophysiology Department, IRCCS Policlinico San Donato, Piazza E. Malan 1, 20097 San Donato Milanese, Milano, Italy; Arrhythmia and Electrophysiology Department, IRCCS Policlinico San Donato, Piazza E. Malan 1, 20097 San Donato Milanese, Milano, Italy; Arrhythmia and Electrophysiology Department, IRCCS Policlinico San Donato, Piazza E. Malan 1, 20097 San Donato Milanese, Milano, Italy; Arrhythmia and Electrophysiology Department, IRCCS Policlinico San Donato, Piazza E. Malan 1, 20097 San Donato Milanese, Milano, Italy; Arrhythmia and Electrophysiology Department, IRCCS Policlinico San Donato, Piazza E. Malan 1, 20097 San Donato Milanese, Milano, Italy; Stem Cells for Tissue Engineering Laboratory, IRCCS Policlinico San Donato, piazza Malan 2, 20097 San Donato Milanese, Milan, Italy; Vita-Salute San Raffaele University, Milan, Italy; Arrhythmia and Electrophysiology Department, IRCCS Policlinico San Donato, Piazza E. Malan 1, 20097 San Donato Milanese, Milano, Italy; Arrhythmia and Electrophysiology Department, IRCCS Policlinico San Donato, Piazza E. Malan 1, 20097 San Donato Milanese, Milano, Italy; Arrhythmia and Electrophysiology Department, IRCCS Policlinico San Donato, Piazza E. Malan 1, 20097 San Donato Milanese, Milano, Italy; Arrhythmia and Electrophysiology Department, IRCCS Policlinico San Donato, Piazza E. Malan 1, 20097 San Donato Milanese, Milano, Italy; Arrhythmia and Electrophysiology Department, IRCCS Policlinico San Donato, Piazza E. Malan 1, 20097 San Donato Milanese, Milano, Italy; Arrhythmia and Electrophysiology Department, IRCCS Policlinico San Donato, Piazza E. Malan 1, 20097 San Donato Milanese, Milano, Italy; Arrhythmia and Electrophysiology Department, IRCCS Policlinico San Donato, Piazza E. Malan 1, 20097 San Donato Milanese, Milano, Italy; Arrhythmia and Electrophysiology Department, IRCCS Policlinico San Donato, Piazza E. Malan 1, 20097 San Donato Milanese, Milano, Italy; Arrhythmia and Electrophysiology Department, IRCCS Policlinico San Donato, Piazza E. Malan 1, 20097 San Donato Milanese, Milano, Italy; Arrhythmia and Electrophysiology Department, IRCCS Policlinico San Donato, Piazza E. Malan 1, 20097 San Donato Milanese, Milano, Italy; Arrhythmia and Electrophysiology Department, IRCCS Policlinico San Donato, Piazza E. Malan 1, 20097 San Donato Milanese, Milano, Italy; Stem Cells for Tissue Engineering Laboratory, IRCCS Policlinico San Donato, piazza Malan 2, 20097 San Donato Milanese, Milan, Italy; Stem Cells for Tissue Engineering Laboratory, IRCCS Policlinico San Donato, piazza Malan 2, 20097 San Donato Milanese, Milan, Italy; Clinical Genomics – SMEL, IRCCS San Raffaele Hospital, Milan, Italy; Vita-Salute San Raffaele University, Milan, Italy; Clinical Genomics – SMEL, IRCCS San Raffaele Hospital, Milan, Italy; School of Medicine and Surgery, University of Milano-Bicocca, Monza, Italy; Vita-Salute San Raffaele University, Milan, Italy; Clinical Genomics – SMEL, IRCCS San Raffaele Hospital, Milan, Italy; Programme in Cardiovascular and Metabolic Disorders and Centre for Computational Biology, Duke-NUS Medical School Singapore, Republic of Singapore; Arrhythmia and Electrophysiology Department, IRCCS Policlinico San Donato, Piazza E. Malan 1, 20097 San Donato Milanese, Milano, Italy; Vita-Salute San Raffaele University, Milan, Italy

**Keywords:** Brugada syndrome, Epicardial arrhythmogenic substrate, Genotype, Phenotype, Predictors, SCN5A

## Abstract

**Aims:**

Brugada syndrome (BrS) is associated with an increased risk of sudden cardiac death due to ventricular tachycardia/fibrillation (VT/VF) in young, otherwise healthy individuals. Despite SCN5A being the most commonly known mutated gene to date, the genotype–phenotype relationship is poorly understood and remains uncertain. This study aimed to elucidate the genotype–phenotype correlation in BrS.

**Methods and results:**

Brugada syndrome probands deemed at high risk of future arrhythmic events underwent genetic testing and phenotype characterization by the means of epicardial arrhythmogenic substrate (AS) mapping, and were divided into two groups according to the presence or absence of SCN5A mutation. Two-hundred probands (160 males, 80%; mean age 42.6 ± 12.2 years) were included in this study. Patients harbouring SCN5A mutations exhibited a spontaneous type 1 pattern and experienced aborted cardiac arrest or spontaneous VT/VF more frequently than the other subjects. SCN5A-positive patients exhibited a larger epicardial AS area, more prolonged electrograms and more frequently observed non-invasive late potentials. The presence of an SCN5A mutation explained >26% of the variation in the epicardial AS area and was the strongest predictor of a large epicardial area.

**Conclusion:**

In BrS, the genetic background is the main determinant for the extent of the electrophysiological abnormalities. SCN5A mutation carriers exhibit more pronounced epicardial electrical abnormalities and a more aggressive clinical presentation. These results contribute to the understanding of the genetic determinants of the BrS phenotypic expression and provide possible explanations for the varying degrees of disease expression.

**Figure:**
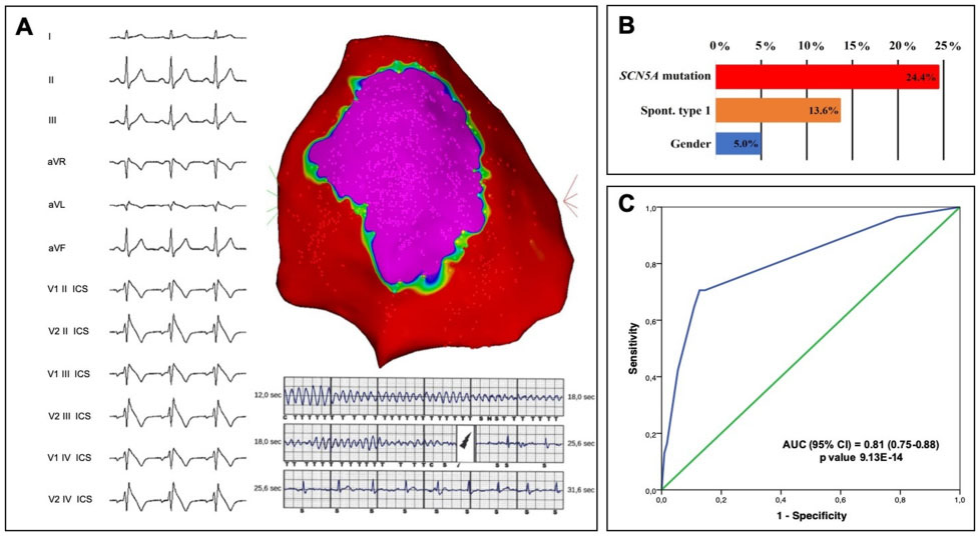

**See page 1091 for the editorial comment on this article (doi: 10.1093/eurheartj/ehaa994)** 

## Introduction

Since its description, the Brugada Syndrome (BrS) has gained increased scientific interest as a cause of sudden cardiac death (SCD) due to ventricular tachycardia/fibrillation (VT/VF) in young and otherwise healthy individuals.^1^ Brugada syndrome is believed to be a genetic disease, although the majority of clinically confirmed cases lack molecular validation, due to our current lack of understanding of the genetics of this syndrome.^2^ While more than 20 genes have been considered as causative of this syndrome and included in diagnostic panels,^3^ the disease causal role of all these genes, except *SCN5A*, has been recently challenged, citing the lack of systematically obtained evidence.^3^

Variants in the *SCN5A* gene are found in ∼21% of all BrS probands.^4^ The current limited knowledge about the genetic basis of BrS prevents the expansion of the diagnostic panel to include genes other than *SCN5A.* ^3^ It is imperative to understand the genetics of this syndrome before using molecular validation for decision-making about patient care.

Many people with the syndrome are completely unaware, as they can live for decades, or even their whole life, without symptoms. However, the first manifestation of the syndrome can be unexpected SCD, and genetic testing cannot always identify those at risk.

Currently, the use of implantable cardioverter-defibrillators (ICD) is the main therapeutic option for high-risk patients.^1^ ^5^ Recent pioneering studies have identified a discrete arrhythmogenic substrate (AS) located in the epicardium of the right ventricle, which is related to both the type 1 electrocardiogram (ECG) pattern and ventricular arrhythmia (VA) inducibility, thus representing a key phenotypic expression of the disease. Accordingly, the presence and electrophysiological properties of the AS likely have a crucial role in the clinical manifestation, alongside the natural history of BrS.^6–8^

To date, in the setting of BrS, there is a lack of information regarding genotype–phenotype association. Therefore, in the present study, we sought to evaluate the role of current genetic testing among a large series of BrS probands at risk of SCD.

## Methods

### Study population

Full details of the rationale and design of the BrS registry have been previously published (NCT02641431; NCT03106701).^7–9^ All consecutive BrS probands referred to the Arrhythmology Department of IRCCS Policlinico San Donato undergoing both genotype and epicardial phenotype assessment have been prospectively enrolled. Medical history, physical examination, baseline ECG, and signal-averaged ECG (SAECG) were obtained in all patients. All patients enrolled had an ICD implanted, as they were deemed as high risk. Further methodological details are provided in the Supplementary material online.

The protocol was reviewed and approved by the local Institutional Ethics Committee, and all participants provided written informed consent, in compliance with the Declaration of Helsinki. All authors had full access to all data in the study and take responsibility for its integrity and data analysis.

### Electrophysiological study and mapping procedure

Electrophysiological study (EPS) was systematically performed as previously described (Supplementary material online, *Methods section*).^8^ All patients underwent a combined endo-epicardial mapping procedure using a three-dimensional (3D) mapping system (CARTO 3, Biosense Webster, CA, USA). Further details are provided in the Supplementary material online. All maps were obtained at baseline conditions and after drug challenge (ajmaline up to 1 mg/kg in 5 min). The abnormal electrograms (EGMs) were identified if they met at least one of the following characteristics: (i) a wide duration (>110 ms) with fragmented component (>3 distinct peaks); (ii) late component of low-voltage amplitude ranging from 0.05 to 1.5 mV; (iii) distinct and delayed component exceeding the end of the QRS complex; and (iv) discrete double activity. Total signal duration was measured for each potential before and after drug challenge as previously described.^7^

The potential duration map was created by collecting the duration of each EGM. As a result, a colour-coded map was obtained showing the regions displaying the shortest (red colour) and the longest (purple colour) durations. Arrhythmogenic substrate areas were measured and validated by two expert electrophysiologists using CARTO3 system, both blinded to the genetic analysis conducted at a different institution.

### Genetic testing

All patients were screened for *SCN5A* gene mutations using genomic DNA and processed with Next Generation Sequencing (TruSight One sequencing kit with NextSeq platform), performed at San Raffaele Hospital. We used Varsome to get an ACMG classification and included all variants with pathogenic (P)/likely pathogenic (LP) classification but excluded benign (B)/likely benign (LB) and variants of unknown significance (VUS).^10^

### Statistical analysis

Data were analysed by Kruskal–Wallis test followed by Dunn’s multiple comparisons or one-way ANOVA followed by Tukey’s test, or chi-square test, as appropriate. Linear regression analysis was used to select variables that predict quantitative variation in the size of the AS. Logistic regression analysis was used to predict a large AS size (≥6.3 cm^2^) on the basis of the set of predictor variables identified in the linear regression model. This cut-off was chosen according to the median value of the substrate size found in the present study population. Receiver-operating characteristic (ROC) curve was constructed to evaluate the performance of the variables gathered from the linear regression model in predicting a large AS size (≥6.3 cm^2^), and the area under the curve and its statistical significance were calculated.

Statistical significance was defined as *P*-value <0.05 unless otherwise indicated. Statistical analyses were conducted using SPSS (v.23, IBM SPSS Statistics). Further detailed statistical analyses are described in the Supplementary material online.

## Results

### Study population characteristics

Among 201 patients, 195 probands (156 males, 80%; mean age 42.7 ± 12.2 years) were included in this study. Six patients harboured an LB and VUS variant and then were excluded (Supplementary material online, *Table S5*). Among the enrolled patients, 23 (11.8%) survived a previous cardiac arrest, and 75 (38.5%) had documented appropriate ICD therapies due to VAs. Forty-three subjects (22.1%) presented with a spontaneous type 1 pattern, 55 (28.2%) had family history of sudden death in relatives before the age of 40 years old, and 93 (47.7%) were inducible for VT/VF at the EPS. Overall clinical, anatomical, and electrophysiological characteristics are summarized in *Table 1*. Forty-nine (49/195, 25.1%) were found to harbour an *SCN5A* mutation. Clinical and electrophysiological characteristics comparing *SCN5A* mutation positive and negative patients are described in *Table 1*.

**Table 1:** Clinical, anatomical, and electrophysiological characteristics of the study population

	Overall (*n* = 195)	*SCN5A* mutation+ (*n* = 49)	*SCN5A* mutation− (*n* = 146)	*P-*value
Male, *n* (%)	156 (80)	38 (77.6)	118 (71.9)	0.681
Age (years) (mean ± SD)	42.7 ± 12.2	40.9 ± 11.3	43.4 ± 12.5	0.124
Spontaneous type 1 pattern, *n* (%)	43 (22.1)	16 (32.7)	27 (18.5%)	0.047
Family history of SD, *n* (%)	55 (28.2)	14 (28.6)	41 (28.1)	1.000
Aborted cardiac arrest, *n* (%)	23 (11.8)	11 (22.4)	12 (8.2)	0.018
Syncope, *n* (%)	79 (41.6)	27 (55.1)	52 (35.6)	0.030
Spontaneous VT/VF requiring ICD therapy, *n* (%)	75 (38.5)	26 (53.1)	49 (33.6)	0.018
Inducible VT/VF at EPS, *n* (%)	93 (47.7)	22 (44.9)	71 (48.6)	0.741
Previous atrial tachyarrhythmias				
Atrial fibrillation, *n* (%)	50 (25.8)	21 (43.8)	29 (19.8)	0.002
Atrial flutter, *n* (%)	14 (7.2)	3 (6.1)	11 (7.5)	1.000
Previous AVNRT, *n* (%)	37 (19)	5 (10.2)	32 (21.9)	0.092
PQ interval, ms (mean ± SD)	179.9 ± 30.5	202.1 ± 33.1	172.6 ± 25.8	<0.001
QRS duration ≥120 ms, *n* (%)	45 (23.1)	21 (42.9)	24 (16.4)	<0.001
f-QRSd (mean ± SD)	114.4 ± 15.9	122.2 ± 19.0	111.8 ± 13.8	0.001
RMS40 (mean ± SD)	19153.7 ± 15894.7	14312.4 ± 13410.4	20778.7 ± 16367.6	0.013
LAS40 (mean ± SD)	43.4 ± 14.5	49.5 ± 18.4	41.3 ± 12.3	0.005
Arrhythmogenic substrate characteristics				
Baseline substrate size (cm^2^) (mean ± SD)	6.3 ± 3.2	9.0 ± 3.8	5.3 ± 2.4	<0.001
Substrate size after ajmaline (cm^2^) (mean ± SD)	13.6 ± 5.9	18.8 ± 5.7	11.9 ± 4.8	<0.001
Baseline potential duration (ms) (mean ± SD)	108.2 ± 40.1	127.9 ± 46.0	101.6 ± 35.6	<0.001
Potential duration after ajmaline (ms) (mean ± SD)	202.6 ± 28.4	220.5 ± 31.5	196.7 ± 24.7	<0.001

AVNRT, atrio-ventricular node re-entrant tachycardia; BrS, Brugada syndrome; ECG, electrocardiogram; EPS, electrophysiological study; ICD, implantable cardioverter-defibrillator; LAS, duration of low-amplitude signals <40 µV; RMS, root mean square voltage of the terminal 40 ms of the filtered QRS complex; SD, standard deviation; SD, sudden death; SVT, supraventricular arrhythmias; VT/VF, ventricular tachycardia/fibrillation.

### Clinical presentations

*Table 1* shows the clinical characteristics of the study cohort according to the genetic status. Patients harbouring *SCN5A* mutations more frequently presented with a spontaneous type 1 pattern as compared with *SCN5A* mutation-negative patients. Compared with those without, *SCN5A* mutation positives more frequently experienced a cardiac arrest, spontaneous life-threatening VAs, and syncope episodes. Furthermore, *SCN5A* carriers more frequently exhibited conduction disturbances (atrio-ventricular and in the right or left bundle; *Table 1*). Atrial fibrillation was more frequently documented in the *SCN5A* mutation-positive group. Other clinical factors, such as gender, age, family history of sudden death, and inducibility for VT/VF during the EPS, were not significantly different between the groups. Non-invasive SAECG was successfully assessed in all patients. By SAECG analysis, patients with an *SCN5A* mutation exhibited more abnormalities. The values of f-QRSd, RMS40, and LAS40 are shown in *Table 1*.

### Arrhythmogenic substrate characterization

**Figure 1:**
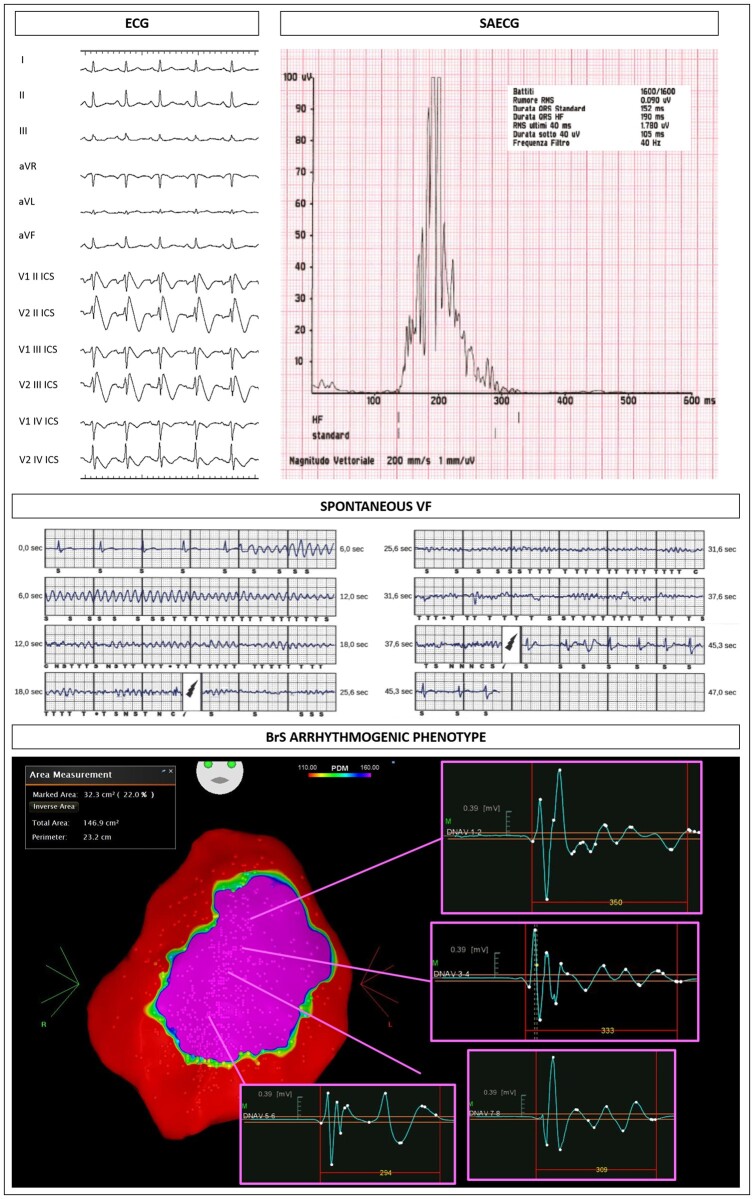
Exemplary case of an *SCN5A* carrier with a spontaneous type 1 pattern and abnormal late potentials at the signal-averaged electrocardiogram (top panel). Middle panel shows one of the appropriate shocks by the implantable cardioverter-defibrillators, implanted in primary prevention. The epicardial mapping demonstrated a large area of electrical abnormalities (32.3 cm^2^), representing the Brugada syndrome arrhythmogenic substrate (purple colour in CARTO map).

**Figure 2:**
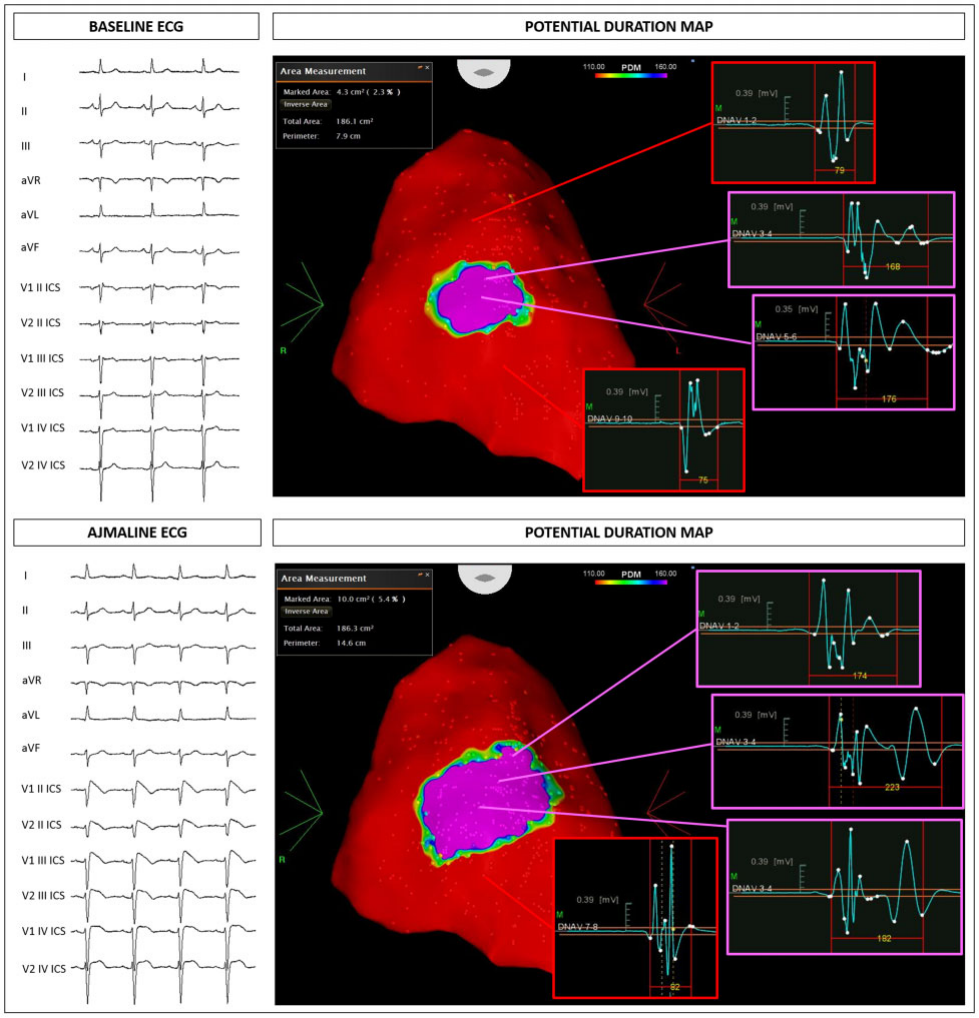
Example of a patient negative to the genetic test. Baseline epicardial mapping demonstrated a 4.3 cm^2^ area of abnormal electrograms (top panel), increasing to 10 cm^2^ after ajmaline.

The size of the AS both at baseline and after ajmaline challenge was significantly larger in patients harbouring an *SCN5A* mutation (*Table 1*). Representative examples of the ECG and epicardial AS are shown for patients harbouring mutations in *SCN5A* (*Figure 1* and Supplementary material online, *Figures S1* and *S2*) and for an *SCN5A* mutation-negative patient (*Figure 2*). Electrophysiological characteristics are summarized in *Table 1*. The AS extent and the potential duration at baseline and after ajmaline administration were significantly increased in patients with *SCN5A* mutation (*Table 1*). Individual variants and their respective substrate areas and potential durations are shown in Supplementary material online, *Table S5*. Electrophysiological parameters for missense variants compared with non-missense variants are displayed in Supplementary material online, *Table S6*.

### Predictors of the arrhythmogenic substrate

**Figure 3:**
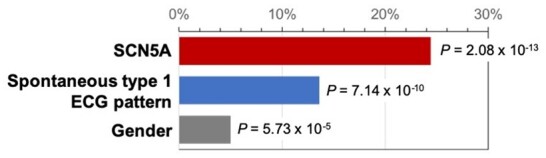
Proportion of variance explained in arrhythmogenic substrate size by each variable included in the linear regression model. The level of statistical significance is also indicated for each variable. SCN5A mutation explained 24.4% of the variance in arrhythmogenic substrate (*F*_change_ = 62.4), spontaneous type 1 pattern 13.6% (*F*_change_ = 42.1), and male gender 5.0% (*F*_change_ = 16.6).

The following predictors were included at each step of the regression model since they significantly contributed to explain the size of the AS: *SCN5A* mutation carriers (*F*_change_ = 62.4, *P* = 2.08E−13), spontaneous type 1 ECG pattern (*F*_change_ = 42.2, *P* = 7.14E−10), and gender (*F*_change_ = 16.6, *P* = 6.72E−5) (*Figure 3* and Supplementary material online, *Table S1*). The final model explained ∼43% of the quantitative variation in the size of the AS, most of which was accounted for by the presence of an *SCN5A* mutation (variance explained ∼24%) and the manifestation of a spontaneous type 1 ECG pattern (variance explained ∼14%) (*Figure 3* and *Take-home figure*). The effect of SCN5A mutation alone was independent from spontaneous type 1 status, as demonstrated by the linear regression analysis (Supplementary material online, *Results* and *Figure S3*). Furthermore, patients carrying an *SCN5A* mutation have 14 times the odds of having an AS ≥6.3 cm^2^ (95% CI 5.6–35.8) (Supplementary material online, *Table S2*). In addition, the presence of a spontaneous type 1 ECG pattern and male gender significantly predicted a large substrate size (see Methods section and Supplementary material online, *Methods* and *Tables S2* and *S3*). An ROC curve was constructed based on the variables retained in the logistic regression model (carrying *SCN5A* mutation, presence of a spontaneous type 1 ECG pattern, and gender), showing an area under the curve of 0.811 (95% CI 0.748–0.875, *P* = 9.13E−14; *Take-home figure*).

**Take-home figure:**
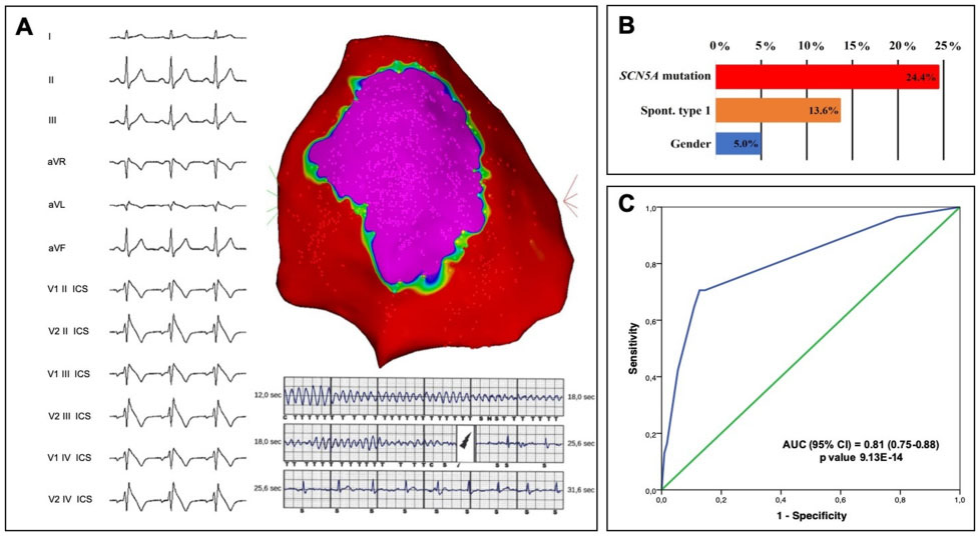
Brugada syndrome phenotypic expression predictors. (*A*) Large Brugada syndrome epicardial substrate in a male patient with spontaneous type 1 electrocardiogram pattern experiencing appropriate implantable cardioverter-defibrillator therapy. (*B*) Specific contribution of each variable (*SCN5A* mutations, spontaneous type 1 electrocardiogram pattern, and gender), in explaining the variance of the arrhythmogenic substrate. (*C*) Receiver-operating characteristic curve analysis demonstrating the accuracy of the model for the prediction of a large arrhythmogenic substrate (≥6.3 cm^2^).

## Discussion

This study provides novel insights into the relationship between the genotype and the phenotype expression in patients with BrS. The main findings of this study are (i) the genotype predicts the BrS phenotypic expression and (ii) patients harbouring an *SCN5A* mutation exhibit a larger epicardial AS area and more prolonged EGMs.

### Brugada syndrome genotype

Since its first description nearly 30 years ago, BrS has gained a significant interest due to the risk of SCD in young and otherwise healthy individuals.^11^ This has prompted active research to understand the genetic mechanisms unveiling the role of the sodium channel in the syndrome.^12–15^ The *SCN5A* gene, encoding the pore-forming alpha-subunit of the cardiac sodium channel, has been the most studied gene thus far due to its undeniable association with BrS,^2^ generally accepted to result because of a loss of function of the sodium channel.^16^ ^,^ ^17^ In a recent meta-analysis including 1780 BrS patients of both Asian and Caucasian descent,^18^ patients with *SCN5A* mutations had a younger age at onset of symptoms and a higher rate of the spontaneous type 1 BrS pattern. Additionally, *SCN5A* patients exhibited more pronounced electrophysiological abnormalities and had a worse prognosis in terms of life-threatening arrhythmias in both Asian and Caucasian patients.

In the present report of 195 BrS probands, we found that *SCN5A* mutation carriers had a more aggressive clinical manifestation as compared with patients without an *SCN5A* mutation, consistent with previous studies that reported an increase in conduction abnormalities on ECG and a higher risk for cardiac events in patients harbouring *SCN5A* mutations.^19^ ^,^ ^20^ In fact, as demonstrated in the present study, carrying an *SCN5A* mutation contributed to ∼26% of the variation in the AS. However, as aborted cardiac arrests and spontaneous life-threatening arrhythmias still occurred at a rate of ∼8% and 34%, respectively, in the *SCN5A* mutation*-*negative group, it is evident that this parameter cannot be considered the only marker of risk for sudden death in this population. This concept may support the hypothesis that BrS could also be an oligo or polygenic disorder, and that multiple variants might influence the phenotype by affecting the function of the channel, and they may also predispose to the disease, in association with various environmental factors.^21^ This could, at least partially, explain the variable expressivity and the incomplete penetrance that characterizes the BrS phenotype.

When analysing *SCN5A* variants according to their Varsome predicted pathogenicity (P/LP vs. VUS) or topographic location of the variant (transmembrane vs. not), no statistically significant differences could be observed, probably due to a relatively small cohort of patients for this type of analysis (Supplementary material online, *Tables S7* and *S8*). Finally, a higher rate of spontaneous type 1 and a larger baseline substrate size were frequently observed among patients with a non-missense *SCN5A* mutation. This may be due to the inclusion of mutations resulting in a truncated protein with no sodium current at the membrane in the non-missense group, which may be associated with a severe phenotype. However, substrate size after ajmaline was not significantly different among the two groups, indicating similar functional abnormalities.

### Brugada syndrome phenotype

After initial reports of electrophysiological abnormalities in the epicardium of the right ventricle in BrS patients, it has been demonstrated that these electrophysiological abnormalities are mechanistically responsible for the type 1 BrS ECG pattern, and their successful elimination by catheter ablation may result in the abolition of the BrS pattern and suppression of VAs.^8^ ^,^ ^22^ Furthermore, patients experiencing symptoms (syncope, aborted cardiac arrest, or spontaneous life-threatening VT/VF) more frequently show aggressive epicardial electrophysiological abnormalities compared with asymptomatic subjects.^7–9^

A study by Nademanee *et al.* ^23^ demonstrated an increase in collagen deposition and subtle fibrosis in the right ventricular outflow tract of BrS patients. Furthermore, inflammation and apoptosis have been reported in myocardial biopsies of the right ventricle.^24^ In our initial experience regarding BrS ablation, large areas of low voltage (bipolar signal <1.5 mV) were demonstrated in the epicardium of patients with a severe clinical phenotype.^25^ It could not be ruled out that these low-voltage epicardial regions may result from structural abnormalities, such as fibrosis, especially in cases with a malignant phenotype. The latter may also explain the nature of fragmented and delayed potentials that could be determined by local epicardial slow conduction due to functional derangements and/or increased coupling resistance.^26–29^ Furthermore, one cannot exclude that phase 2 reentry^30^ may represent one of the mechanisms of VAs in the absence of clear slow conduction and in presence of a suitable substrate.

In the present study, spontaneous type 1 pattern contributed ∼14% of the variation in the AS. These observations may support the concept that the epicardial substrate represents the ‘true’ BrS phenotype, characterized by the epicardial abnormalities, rather than the ECG manifestation, which is transient.

Furthermore, the noteworthy feature of this study is the independent association between the extent of the electro-anatomical AS and the genetic status of the disease, as demonstrated by the multivariate analysis. In fact, as shown in our series, a multivariable model including the presence of an *SCN5A* mutation, spontaneous type 1 ECG pattern, and male gender predicted a substrate area ≥6.3 cm^2^.

These findings further strengthen the relevance of genetic evaluation, as genetics clearly contribute to the electrophysiological phenotypic expression of the disease, and this could enable more individualized management. These concepts support the evidence that the epicardial substrate may be the pathological consequence of altered genetics and environmental factors, resulting in differences in the phenotypic expression and clinical manifestation of the disease. The sodium channel impairment seems to affect the epicardial conduction properties of the right ventricle. The *SCN5A* mutation determines a lower sodium current directly by modifying the protein structure. However, the use of sodium channel blockers to unmask the epicardial abnormalities also in patients without *SCN5A* mutations may suggest the presence of subtle impairments in the sodium channel function, and ajmaline may magnify the extent of such abnormalities. Furthermore, there could be other genetic variants that may affect sodium channel function as the Nav1.5 protein could be post-translationally modified by signalling pathways.

Therefore, current genetic testing alone cannot explain the complexity of this disease, and this supports pursuing more studies in this field to improve the knowledge regarding the genetic ‘milieu’ behind the AS and the genetic modulators influencing the disease penetrance and manifestation.

These results have important implications, providing insight into how an *SCN5A* mutation is associated with an increased risk of life-threatening arrhythmias.^19^ The genetic assessment may be used as a tool to non-invasively predict the presence of potentially dangerous properties of the substrate that lead to the increased arrhythmogenic risk in this population, and these findings may help clinicians in refining patients’ risk stratification.

The present report provides, for the first time, novel insights regarding the relationship between the genotype and the electrophysiological parameters in a large cohort of BrS probands. In this study, patients harbouring *SCN5A* mutations showed more aggressive electrophysiological epicardial abnormalities as compared with those without *SCN5A* mutations, which may explain why these patients have a more severe clinical presentation, more frequently experiencing cardiac arrest or VAs.^19^

In the present patient population, *SCN5A*-positive patients also more frequently exhibited ventricular conduction disturbances as well as abnormal late activity in SAECG. It has been demonstrated that the arrhythmic substrate can be considered their main electrophysiological cause.^9^ Moreover, non-invasive LPs are related to a prominent arrhythmic substrate area, and they have been used to detect individuals at high risk of future life-threatening arrhythmias.^31^ In the present study, the association between abnormal LPs and the larger arrhythmic substrate in patients harbouring *SCN5A* mutations further strengthens the concept of an increased arrhythmic risk in this population.

### Limitations

This is the first prospective study evaluating the correlation between genotype and phenotype in BrS in the form of the epicardial AS in a large cohort of BrS probands. This study was conducted in a population with various clinical characteristics, evaluated and/or referred to an experienced BrS centre, because they showed an increased arrhythmic risk profile. Nevertheless, as nearly half of the patients did not experience appropriate ICD therapies at the time of this study, we acknowledge that this population may have heterogeneous clinical characteristics with different arrhythmic risk profiles. Therefore, these results might be not applicable to other patient populations. This may also explain the lack of differences in inducibility rate according to genetic status. These results cannot be used to draw conclusions regarding the usefulness of EPS for risk stratification.

The role of SCN5A mutation type, pathogenicity prediction, and topographic location of the variant will be matter of future research, as the relatively limited sample size of this study prevents the drawing of significant conclusions in this regard. Furthermore, what role genes other than *SCN5A* may play remains unknown.

## Conclusions

These results demonstrate that the genetic background is the main determinant of the epicardial electrophysiological abnormalities, contributing to the understanding of the BrS phenotypic expression, providing possible explanations for the varying degrees of disease manifestation. Patients harbouring *SCN5A* mutations exhibit more pronounced epicardial electrical abnormalities, including a more extensive AS and more aggressive clinical presentation.

## Supplementary material

Supplementary material is available at *European Heart Journal* online.

## Funding

This study was partially supported by Ricerca Corrente funding from Italian Ministry of Health to IRCCS Policlinico San Donato.

## Data availability

The data underlying this article are available from the corresponding author on reasonable request.

**Conflict of interest:** none declared.

## Supplementary Material

ehaa942_Supplementary_Data
