# Supplementary material for: Brugada syndrome genetics is associated with phenotype severity
Source: Eur Heart J. 2020 Nov 21;42(11):1082–90. doi: 10.1093/eurheartj/ehaa942 (PMC7955973; doi:10.1093/eurheartj/ehaa942)

**Online Data Supplement**

## **METHODS**

## **Patient population and Definitions**

All patients were evaluated or referred to the Arrhythmology Department of IRCCS Policlinico San Donato, which is a high-volume tertiary center for BrS management.

This study was conducted in a population with relatively heterogeneous clinical characteristics, evaluated and/or referred to our tertiary center because they were deemed high-risk patients. All of them had an ICD implanted. Nearly half of the present cohort experienced potentially life-threatening arrhythmic events or previous cardiac arrest. Although at the time of this study the remaining patients did not receive appropriate ICD therapies (116/195, 59.5%), they presented with clinical features that suggested an increased arrhythmic risk: history of syncope [45/116, 38.8%], spontaneous type 1 [14/116, 12.1%], early family history of sudden death – before 40 years of age [37/116, 31.9%], sinus node dysfunction [6/116, 5.2%], SCN5A mutation [21/116, 18.1%], inducible VT/VF during EPS [28/116, 24.1%], SAECG abnormalities [75/116, 65%], self-terminating VAs in ICD memory [22/116, 19%].

Brugada syndrome was diagnosed in the presence of a coved-type ST elevation of ≥ 2 mm, as documented in ≥ 1 lead from V1 to V3 positioned in the second, third, or fourth intercostal space. Because of the variable nature of the BrS-ECG pattern, BrS patients were classified according to their ECG at the time of presentation, defined as the spontaneous ECG type pattern. ECG pattern definitions were based on the second consensus statement regarding Brugada Syndrome.

**Arrhythmogenic substrate mapping**

None of the patients had prior mapping or ablation procedures. After having performed [endocardial](https://www.sciencedirect.com/topics/medicine-and-dentistry/endocardium) (Endo) electroanatomical mapping of the [right ventricle](https://www.sciencedirect.com/topics/medicine-and-dentistry/right-ventricle) (RV), aimed at delimitation of RV boundaries, epicardial (Epi) access was obtained using fluoroscopy-guided subxyphoidal [puncture](https://www.sciencedirect.com/topics/medicine-and-dentistry/puncture), and a steerable sheath (Agilis EPI, Abbott, MN, USA) was introduced. Detailed RV Endo-Epi mapping was performed using a 3-dimensional (3D) mapping system (CARTO 3, Biosense Webster, CA, USA) with a high-density mapping catheter (DecaNAV, Biosense Webster; 1 mm electrodes with 2-8-2 interelectrode spacing).

All Epi-maps were obtained at baseline conditions and after drug challenge (ajmaline 1 mg/kg in 5 min). Ajmaline was administered (1) to achieve the maximal ST-segment elevation and unmask, if necessary, the type 1 ECG pattern and (2) to identify the real extent of the regions displaying fragmented and abnormal electrograms (EGMs). All the potential duration maps (PDMs) were performed by collecting the duration of each bipolar EGM. Bipolar EGMs were filtered from 16 Hz to 500 Hz, displayed at 200 mm/s speed, and were recorded between the distal electrode pair. EGM acquisition was performed only if the multipolar catheter was stable in each epicardial position and if the EGM morphology, evaluated by the operators, was consistent and repetitive for at least 3 consecutive beats, thus avoiding artifacts. Acquisition was excluded if the technical quality was insufficient (noise due to catheter movement or poor catheter contact).

None of the study subjects were taking any antiarrhythmic, antipsychotic, or other drugs known to have a significant effect on cardiac ion channels at the time of SAECG recording.

We categorized patients according to late potentials (LP) abnormalities recorded with signal averaged ECG. The parameters assessed by computer algorithm were: (i) filtered QRS duration (f-QRSd); (ii) root mean square voltage of the terminal 40 ms of the filtered QRS complex (RMS40); and (iii) duration of low-amplitude signals <40 µV of QRS in the terminal filtered QRS complex (LAS40)^4^.

**Statistical analysis**

Data were analyzed by Kruskal-Wallis test followed by Dunn’s multiple comparisons test or one-way ANOVA and Tukey post-hoc test, or chi-squared test, as appropriate. Linear regression analysis was used to select variables that predict quantitative variation in the size of the arrhythmogenic substrate. We used a forward stepwise regression approach, where at each step of the variable selection, the predictor variable not in the equation that has the smallest probability of F is entered (significance of F change, p < 0.05) and variables already in the regression equation are removed (significance of F change, p > 0.1). Among variables that were tested in the model, multicollinearity statistics were evaluated by means of tolerance and variance inflation factor (VIF) values. The variables included in the final linear regression model had tolerance values between 0.971 and 0.991 and VIF values between 1.009 and 1.030, reassuring of low multicollinearity between the selected variables. In order to ensure that the asymptotic estimates in the linear regression model were not affected by the presence of outliers and potential lack of homoscedasticity, we also derived bootstrap estimates, 95% CI and significance tests for the regression coefficients of each predictor variable selected in the linear model. Bootstrap estimates of coefficients supported the asymptotic estimates in the linear regression model and are reported in Supplemental Table 1.

Logistic regression analysis was used to predict a large arrhythmogenic substrate size (≥ 6.3 cm^2^) on the basis of the set of predictor variables identified in the linear regression model. This cut-off was chosen according to the median value of the substrate size found in the present study population. Therefore, the dependent variable is a binary classifier of BrS patients (n=195) with the size of the arrhythmogenic substrate as follows: 0 = size of the arrhythmogenic substrate <6.3 (n=110) and 1 = size of the arrhythmogenic substrate ≥ 6.3 (n=85). Similar to the linear regression model, to build the logistic regression model, we used a step forward variable selection approach, where at each step a predictor variable is entered in the model based on the significance of the score statistic (p < 0.05). Variables tested in the model included gender, spontaneous type 1 pattern, *SCN5A* mutation carriers, and mutations in other diagnostic panel genes. Bootstrap estimates of coefficients supported the asymptotic estimates in the linear regression model and are reported in Supplemental Table 3. The final model fit statistics indicated adequate fit: goodness-of-fit χ2 (n=195, df 3) = 72.2 (p = 1.6E-15); Cox and Snell *R*^2^ = 0.309; Nagelkerke *R*^2^ = 0.414; Hosmer and Lemeshow Test χ2 (df 4) = 3.13 (p = 0.537). Logistic regression coefficients were also used to estimate odds ratios (ORs) for each of the independent variables in the model. A receiver-operating characteristic curve for substrate size and the significant variables in the logistic regression model was generated, and the area under the curve and its statistical significance were calculated. Statistical significance was defined as p-value < 0.05 unless otherwise indicated. Statistical analyses were conducted using SPSS (v.23, IBM SPSS Statistics). Statistical analyses are further described in the supplemental material.

**RESULTS**

**Predictors of the arrhythmogenic substrate.** The dependent variable is the size of the arrhythmogenic substrate, and significant predictive variables included at each step of the model are reported. The final model included the following predictor variables: *SCN5A* mutation+, spontaneous type 1 ECG pattern, and gender. Variables (bold font) added to the model at each sequential step are indicated in Table 2. The final model explained ~42% of the variation in the size of the arrhythmogenic substrate. Adj. R^2^, R^2^ that has been adjusted for the number of predictors in the model. Change in R^2^, represents the increase in the variance explained for when an additional predictor is added to the model. df, degrees of freedom. Estimates of the regression coefficients, statistical significance and upper/lower 95% confidence intervals (CI) of the estimates are also provided. At each step of the model, the change in R^2^ is tested with an F-test, which is referred to as the F change. A significant F change (p < 0.05) means that the variable added in that step significantly improved the prediction. Among *SCN5A* mutation carriers with a spontaneous type 1 ECG pattern have a larger substrate size (1.5-fold) than patients without a spontaneous type 1 ECG pattern (Supplemental Figure 3).

The stepwise regression linear model adds one variable after each other on the basis of each variable contribution to explain the substrate size; therefore in the final model that explains ~ 42% of substrate size, the effect of SCN5A alone (24% of substrate size explained) is independent from spontaneous type 1 ECG status. To confirm the effect of SCN5A is “corrected” for spontaneous type 1 ECG status, we did two separate linear regression analyses for patients without spontaneous type 1 ECG (n=152) or with spontaneous type 1 ECG (n=43). In each case the SCN5A variable significantly explained  the substrate size  as follows: (1) in patients without spontaneous type 1 ECG (n=152), SCN5A significance of effect p = 7.04E-9, variance of substrate size explained by SCN5A = 20%; (2) in patients with spontaneous type 1 ECG (n=43), SCN5A significance of effect p = 8.4E-5, variance of substrate size explained by SCN5A = 32%.

Despite the different sample sizes (n=152 vs n=43), this analysis shows that the effect of SCN5A on the substrate size is significant on both patients groups (with or without type 1 ECG pattern) and therefore the association with SCN5A mutations is independent on whether patients have spontaneous type 1 ECG.

**REFERENCES**

1. Antzelevitch C, Brugada P, Borggrefe M, Brugada J, Brugada R, Corrado D, Gussak I, LeMarec H, Nademanee K, Perez Riera AR, Shimizu W, Schulze-Bahr E, Tan H, Wilde A. Brugada syndrome: report of the second consensus conference: endorsed by the Heart Rhythm Society and the European Heart Rhythm Association. Circulation. 2005 Feb 8;111(5):659-70.
2. Gomes JA, Winters SL, Stewart D, Horowitz S, Milner M, Barreca P. A new noninvasive index to predict sustained ventricular tachycardia and sudden death in the first year after myocardial infarction: based on signal-averaged electrocardiogram, radionuclide ejection fraction and Holter monitoring. J Am Coll Cardiol 1987;10:349 –357.
3. Dinov B, Bode K, Koenig S, Oebel S, Sommer P, Bollmann A, Hindricks G, Arya A. Signal-averaged electrocardiography as a noninvasive tool for evaluating the outcomes after radiofrequency catheter ablation of ventricular tachycardia in patients with ischemic heart disease: reassessment of an old tool. Circ Arrhythm Electrophysiol. 2016;9(9). pii: e003673.
4. Ikeda T, Sakurada H, Sakabe K, Sakata T, Takami M, Tezuka N, Nakae T, Noro M, Enjoji Y, Tejima T, Sugi K, Yamaguchi T. Assessment of noninvasive markers in identifying patients at risk in the Brugada syndrome: insight into risk stratification. J Am Coll Cardiol 2001;37:1628 –1634.

**Supplemental Table 1**. Results of the linear regression model (forward stepwise variable selection). Bootstrap estimates of the regression coefficients (B) and their 95% CI.

| Bootstrap estimates^#^ of the regression coefficients | B | Bias | Std. Error | Sig. (2-tailed) | 95% CI Lower | 95% CI Upper |
| --- | --- | --- | --- | --- | --- | --- |
| (Constant) | 1.557 | 0.024 | 0.943 | 0.105 | -0.315 | 3.405 |
| *SCN5A* mutation positive | 3.354 | -0.003 | 0.479 | <0.001 | 2.420 | 4.289 |
| Spontaneous type 1 BrS ECG pattern | 2.756 | -0.007 | 0.474 | <0.001 | 1.825 | 3.678 |
| Gender | 1.808 | -0.013 | 0.505 | <0.001 | 0.804 | 2.812 |

^#^Results are based on 5,000 bootstrap samples

**Supplemental Table 2**. Results of the logistic regression model. The dependent variable is a binary classifier of BrS patients (n=195) with size of the arrhythmogenic substrate as follows: 0 = size of the arrhythmogenic substrate <6.3 (n=110) and 1 = size of the arrhythmogenic substrate ≥6.3 (n=85). The thresholds correspond to the median of the size of the arrhythmogenic substrate in the BrS cohort. Only the significant variables that contributed to the model fit are reported. Exp(B) coefficients represent the odds ratios (ORs) for the predictors, which were calculated with respect to a reference category as indicated in the table.

| Predictor variable in the model | B | S.E. | Wald χ2 | df | Significance (p-value) | Exp(B) | 95% CI  lower | 95% CI  upper |
| --- | --- | --- | --- | --- | --- | --- | --- | --- |
| *SCN5A* mutation carriers  [reference category 0: *SCN5A mutation negative*] | 2.660 | 0.469 | 32.126 | 1 | 1.4E-08 | 14.291 | 5.597 | 35.847 |
| Spontaneous type 1 BrS ECG pattern  [reference category 0: *no spontaneous type 1 pattern*] | 1.821 | 0.439 | 17.253 | 1 | 3.3E-05 | 6.181 | 2.617 | 14.598 |
| Gender  [reference category 0: *female*] | 1.200 | 0.516 | 5.416 | 1 | 0.020 | 3.321 | 1.209 | 9.124 |

**Supplemental Table 3**. Results of the logistic regression model (forward stepwise variable selection). Bootstrap estimates of the regression coefficients (B) and their 95% CI.

| Bootstrap estimates^#^ of the regression coefficients | B | Bias | Std. Error | Sig. (2-tailed) | 95% CI Lower | 95% CI Upper |
| --- | --- | --- | --- | --- | --- | --- |
| (Constant) | -2.268 | -0.109 | 0.669 | <0.0001 | -3.761 | -1.343 |
| *SCN5A* mutation positive | 2.660 | 0.128 | 0.716 | <0.0001 | 1.830 | 4.060 |
| Spontaneous type 1 pattern | 1.821 | 0.073 | 0.484 | <0.0001 | 1.007 | 2.923 |
| Gender | 1.200 | 0.089 | 0.668 | 0.023 | 0.209 | 2.620 |

^#^Results are based on 10,000 bootstrap samples

**Supplemental Table 4**. Results of the linear regression model (forward stepwise variable selection). The dependent variable is the size of the arrhythmogenic substrate and significant predictive variables included at each step of the model are reported. This table provides estimates of the regression coefficients, statistical significance and upper/lower 95% confidence intervals (CI) of the estimates.

| Estimates of the coefficients | B | S.E. | Standardized B | t | Sig. (2-tailed) | 95% CI Lower | 95% CI Upper |
| --- | --- | --- | --- | --- | --- | --- | --- |
| *SCN5A* mutation positive | 3.354 | 0.412 | 0.450 | 8.134 | 5.17E-14 | 2.541 | 4.168 |
| Spontaneous type 1 pattern | 2.756 | 0.433 | 0.353 | 6.370 | 1.38E-09 | 1.902 | 3.609 |
| Gender | 1.808 | 0.444 | 0.224 | 4.076 | 6.73E-05 | .933 | 2.684 |

**Supplemental Table 5. Substrate areas and potential durations by *SCN5A* variant.** Two patients had a double variant in the *SCN5A* gene, which are reported together with the respective substrate areas and potential durations and their specific variants are marked with an asterisk. The variants with a likely benign and VUS prediction listed in this table have been excluded for the main analysis.

| **SCN5A Variant** | **Amino acid change** | **Swiss-Prot** | **Varsome prediction as of 14 July 2020** | **Mutation type** | **Frequency**  **(according to GnomAD)** | **Baseline substrate size (cm^2^) (mean +/- SD)** | **Substrate size after Ajmaline (cm^2^) (mean +/- SD)** | **Baseline potential duration (ms) (mean +/- SD)** | **Potential duration after Ajmaline (ms) (mean +/- SD)** |
| --- | --- | --- | --- | --- | --- | --- | --- | --- | --- |
| c.4850_4852delTCT | p.Phe1563del | DIV | Likely Pathogenic | Deletion | 1/50186 | 14.2 | 21.3 | 91.9 | 227.5 |
| c.809_812dup | p.Phe272AlafsTer40 | SFR1 | Pathogenic | Insertion | Not known | 9.3 | 14.4 | 160.8 | 160.8 |
| c.1111C>T | p.Gln371Ter | SFR1 | Pathogenic | Nonsense | Not known | 7.1 | 26.2 | 75.6 | 184.4 |
| c.3946C>T | p.Arg1316Ter | DIII | Pathogenic | Nonsense | Not known | 11.5 | 15.2 | 186.9 | 199.2 |
| c.2091G>A | p.Trp697Ter | IDL I - II | Pathogenic | Nonsense | Not known | 14.2 | 18.2 | 234.6 | 234.6 |
| c.4867C>T | p.Arg1623Ter | DIV | Pathogenic | Nonsense | 1/251070 | 8.5 | 17.1 | 90.6 | 166.9 |
| c.4700_4701del | p.Phe1567CysfsTer221 | DIV | Pathogenic | Deletion | Not known | 8.2 | 19.3 | 82.3 | 208.9 |
| c.5830C>T* | p.Arg1944Ter | C term | Pathogenic | Nonsense | 1/82674 | 7.2 | 18.8 | 82.9 | 197.9 |
| c.481G>A* | p.Glu161Lys | DI | Likely Pathogenic | Missense | 1/240992 |  |  |  |  |
| c.4867C>T | p.Arg1623Ter | DIV | Pathogenic | Nonsense | 1/251070 | 8.2 | 17.9 | 179.5 | 179.5 |
| c.4294delA | p.Arg1432GlyfsTer31 | SFR3 | Likely Pathogenic | Deletion | Not known | 7.7 | 17 | 75.6 | 195 |
| c.1140+2T>C | n/a | Not Predictable | Pathogenic | Splicing | Not known | 9.9 | 11.5 | 93.7 | 199.6 |
| c.1890G>A | p.Thr630= | IDL I - II | Likely Pathogenic | Synonym | 1/174574 | 11.7 | 20.3 | 93 | 215.1 |
| c.1921delC | p.Gln641ArgfsTer3 | IDL I -II | Pathogenic | Deletion | Not known | 14.8 | 31.5 | 184.6 | 282 |
| c.3840+1G>A | n/a | Not Predictable | Pathogenic | Splicing | 1/125615 | 11.5 | 12.8 | 98.6 | 212.4 |
| c.535C>T | p.Arg179Ter | DI | Pathogenic | Nonsense | 1/248621 | 18.3 | 25.4 | 146.1 | 278.9 |
| c.2893C>T | p.Arg965Cys | IDL II - III | Likely Pathogenic | Missense | 1/15398 | 8.7 | 24.2 | 76.6 | 225.2 |
| c.481G>A | p.Glu161Lys | DI | Likely Pathogenic | Missense | 1/240992 | 8.5 | 18.5 | 98.4 | 177.2 |
| c.2314G>A | p.Asp772Asn | DII | Likely Pathogenic | Missense | 1/49849 | 0 | 6.3 | 69.5 | 214.6 |
| c.4813G>T | p.Gly1605Cys | IDL III - IV | Pathogenic | Missense | 1/122972 | 10.8 | 17.5 | 168.6 | 242.2 |
| c.4534C>T | p.Arg1512Trp | IDL III - IV | Likely Pathogenic | Missense | 1/17948 | 3.9 | 17 | 104.4 | 195.9 |
| c.4057G>A | p.Val1353Met | SFR3 | Likely Pathogenic | Missense | 1/35926 | 8.2 | 17.9 | 154.6 | 202.5 |
| c.4312C>T | p.Pro1438Ser | SFR3 | Likely Pathogenic | Missense | Not known | 0 | 5.1 | 96.8 | 250.2 |
| c.692C>T | p.Ser231Leu | DI | Likely Pathogenic | Missense | Not known | 8.5 | 24 | 112.3 | 240.6 |
| c.5000T>A | p.Val1667Asp | SFR4 | Likely Pathogenic | Missense | Not known | 12 | 18.5 | 94.4 | 205 |
| c.4501C>G | p.Leu1501Val | IDL III - IV | Likely Pathogenic | Missense | 1/50289 | 8.3 | 18 | 77.7 | 183.5 |
| c.1144C>T | p.Leu382Phe | SFR1 | Likely Pathogenic | Missense | Not known | 4.2 | 17.5 | 210.8 | 210.8 |
| c.2441G>A | p.Arg814Gln | DII | Pathogenic | Missense | 1/41307 | 7.3 | 35 | 72.4 | 238.6 |
| c.5089T>C | p.Phe1697Leu | SFR4 | Likely Pathogenic | Missense | Not known | 10.1 | 11.9 | 102.5 | 163.5 |
| c.1045G>A | p.Asp349Asn | SFR1 | Likely Pathogenic | Missense | 1/82983 | 11.4 | 15.3 | 88.8 | 199.8 |
| c.3673G>A | p.Glu1225Lys | DIII | Likely Pathogenic | Missense | 1/249528 | 8.5 | 19.7 | 91.5 | 202.4 |
| c.2542A>G | p.Ile848Val | SFR2 | Likely Pathogenic | Missense | Not known | 0 | 13.3 | 62.6 | 219 |
| c.733C>A | p.Gln245Lys | DI | Likely Pathogenic | Missense | 1/124734 | 6.1 | 16.2 | 147.2 | 215.6 |
| c.880G>A | p.Val294Met | SFR1 | Likely Pathogenic | Missense | 1/41506 | 5.4 | 15 | 131.5 | 214.5 |
| c.5227G>A | p.Gly1743Arg | SFR4 | Pathogenic | Missense | Not known | 17.3 | 24.2 | 173.8 | 285.4 |
| c.4720G>A | p.Glu1574Lys | DIV | Pathogenic | Missense | Not known | 7.6 | 19.8 | 149.5 | 224.3 |
| c.655C>T | p.Arg219Cys | DI | Likely Pathogenic | Missense | 1/61014 | 10.5 | 15.7 | 95.5 | 214.8 |
| c.4140C>G* | p.Asn1380Lys | SFR3 | Pathogenic | Missense | Not known | 7.3 | 23.5 | 158.2 | 198.3 |
| c.2989G>A* | p.Ala997Thr | IDL II - III | Likely Pathogenic | Missense | 1/12622 |  |  |  |  |
| c.2414C>T | p.Ser805Leu | SFR2 | Likely Pathogenic | Missense | 1/124197 | 8.6 | 18.2 | 144.3 | 244.1 |
| c.481G>A | p.Glu161Lys | DI | Likely Pathogenic | Missense | 1/240992 | 10.5 | 18.4 | 166.2 | 253.3 |
| c.1041C>A | p.Asn347Lys | SFR1 | Likely Pathogenic | Missense | Not known | 6.4 | 18.4 | 81.5 | 252.8 |
| c.5102T>G | p.Met1701Arg | SFR4 | Likely Pathogenic | Missense | Not known | 10.8 | 23.9 | 190.6 | 283.2 |
| c.5129C>T | p.Ser1710Leu | SFR4 | Pathogenic | Missense | 1/62861 | 12.2 | 18.5 | 189.1 | 224.1 |
| c.589G>T | p.Asp197Tyr | DI | Likely Pathogenic | Missense | Not known | 7.6 | 13 | 178.6 | 249.5 |
| c.3917G>A | p.Arg1306His | DIII | Likely Pathogenic | Missense | 1/247002 | 4.8 | 11.4 | 160.2 | 257.2 |
| c.422T>A | p.Ile141Asn | DI | Likely Pathogenic | Missense | 1/245302 | 10.2 | 30.1 | 86.6 | 214.9 |
| c.4894C>T | p.Arg1632Cys | DIV | Likely Pathogenic | Missense | 1/251351 | 9.4 | 25.1 | 198.6 | 214.5 |
| c.2182G>A | p.Val728Ile | DII | Likely Pathogenic | Missense | 1/124629 | 10.2 | 17 | 110.4 | 248.9 |
| c.1144C>T | p.Leu382Phe | SFR1 | Likely Pathogenic | Missense | Not known | 14.4 | 23.7 | 185 | 277.4 |
| c.4516C>A | p.Pro1506Thr | IDL III - IV | Likely Pathogenic | Missense | Not known | 10.1 | 20.3 | 164.5 | 218.5 |
| c.5863A>T | p.Asn1955Tyr | C term | Uncertain | Missense | 1/246198 | 0 | 25.1 | 84 | 213.6 |
| c.6004G>A | p.Ala2002Thr | C term | Uncertain | Missense | 1/32580 | 10.3 | 23.7 | 81.2 | 175.4 |
| c.4437+5G>A | n/a | Not Predictable | Uncertain | Splicing | Not known | 13.2 | 18 | 144 | 272.7 |
| c.86_87delinsTG | p.Ala29Val | N term | Uncertain | Missense | Not known | 7.2 | 6.5 | 87 | 185.2 |
| c.3214_3215delinsTC | p.Glu1072Ser | IDL II -III | Uncertain | Missense | Not known | 8.3 | 15.4 | 66.7 | 172.5 |
| c.2039G>A | p.Arg680His | IDL I - II | Likely Benign | Missense | 1/31401 | 11.4 | 15.4 | 82 | 243.8 |

**Supplemental Table 6.** Clinical, anatomical, and electrophysiological characteristics in patients with missense *SCN5A* variants.

|  | ***SCN5A* missense variant**  **(n = 34)** | **Other *SCN5A* variants**  **(n = 15)** | **P value** |
| --- | --- | --- | --- |
| **Male, n (%)** | 28 (82.4) | 10 (66.7) | 0.225 |
| **Age (years) (mean +/- SD)** | 40.6 ± 11.6 | 41.4 ± 11.0 | 0.656 |
| **Spontaneous type 1 pattern, n (%)** | 8 (23.5) | 8 (53.3) | 0.040* |
| **Family history of SD, n (%)** | 8 (23.5) | 6 (40.0) | 0.240 |
| **Aborted Cardiac Arrest, n (%)** | 8 (23.5) | 3 (20.0) | 0.785 |
| **Syncope, n (%)** | 18 (52.9) | 9 (60.0) | 0.647 |
| **Spontaneous VT/VF requiring ICD therapy, n (%)** | 17 (50.0) | 9 (60.0) | 0.518 |
| **Inducible VT/VF at EPS, n (%)** | 15 (44.1) | 7 (46.7) | 0.869 |
| **Previous Atrial Tachyarrhythmias** |  |  |  |
| Atrial Fibrillation, n (%) | 14 (41.2) | 8 (53.3) | 0.430 |
| Atrial Flutter, n (%) | 2 (5.9) | 1 (6.7) | 0.916 |
| **Previous AVNRT, n (%)** | 1 (2.9) | 4 (26.7) | 0.011* |
| **PQ interval, ms (mean +/- SD)** | 209.6 ± 34.8 | 218.9 ± 26.3 | 0.246 |
| **QRS duration ≥120 ms, n (%)** | 15 (44.1) | 6 (40.0) | 0.788 |
| **f-QRSd (mean +/- SD)** | 123.1 ± 18.0 | 120.1 ± 21.8 | 0.794 |
| **RMS40 (mean +/- SD)** | 14473.8 ± 9549.3 | 13945.3 ± 20035.9 | 0.072 |
| **LAS40 (mean +/- SD)** | 47.4 ± 16.8 | 54.3 ± 21.7 | 0.070 |
| **Arrhythmogenic Substrate Characteristics** |  |  |  |
| **Baseline substrate size (cm^2^) (mean +/- SD)** | 8.2 ± 3.8 | 10.8 ± 3.3 | 0.022* |
| **Substrate size after Ajmaline (cm^2^) (mean +/- SD)** | 18.6 ± 5.9 | 19.1 ± 5.3 | 0.756 |
| **Baseline potential duration (ms) (mean +/- SD)** | 129.2 ± 44.1 | 125.1 ± 51.8 | 0.792 |
| **Potential duration after Ajmaline (ms) (mean +/- SD)** | 225.4 ± 28.9 | 209.5 ± 35.2 | 0.140 |

Abbreviations: AVNRT: atrio-ventricular node reentrant tachycardia; BrS: Brugada Syndrome; ECG: electrocardiogram; EPS: electrophysiologic study; LAS: duration of low-amplitude signals < 40 µV; RMS: root mean square voltage of the terminal 40 ms of the filtered QRS complex; SD: standard deviation; SD: sudden death; SVT: supraventricular arrhythmias; VT/VF: ventricular tachycardia/fibrillation.

**Supplemental Table 7.** Electrophysiological characteristics in patients with *SCN5A* variants according to the pathogenicity status.

|  | **SCN5A P/LP variants**  **(n=49)** | **SCN5A VUS**  **(n=5)** | **P-value** |
| --- | --- | --- | --- |
| **Baseline substrate size (cm^2^) (median and IQR)** | 8.6 (7.3-11-1) | 8.3 (3.6-11.8) | 0.507 |
| **Substrate size after Ajmaline (cm^2^) (median and IQR)** | 18.2 (15.5-22.4) | 15.4 (7.9-20.8) | 0.131 |
| **Baseline potential duration (ms) (median and IQR)** | 110.4 (89.7-167.4) | 83.2 (73.9-83.2) | 0.053 |
| **Potential duration after Ajmaline (ms) (median and IQR)** | 214.9 (199.4-243.2) | 185.2 (173.9-229.5) | 0.152 |

Abbreviations: IQR: inter-quartile range; LP: likely pathogenic variant; ms: milliseconds; P: pathogenic variant; VUS: variant of unknown significance.

**Supplemental Table 8.** Electrophysiological characteristics in patients with and without *SCN5A* variant located in the transmembrane region.

|  | **SCN5A transmembrane**  **(n=27)** | **SCN5A**  **non-transmembrane**  **(n=22)** | **P-value** |
| --- | --- | --- | --- |
| **Baseline substrate size (cm^2^) (mean +/- SD)** | 9.3±3.6 | 8.7±4.1 | 0.582 |
| **Substrate size after Ajmaline (cm^2^) (mean +/- SD)** | 19.7±6.1 | 17.5±5.0 | 0.180 |
| **Baseline potential duration (ms) (mean +/- SD)** | 127.9±46.9 | 128.0±46.0 | 0.994 |
| **Potential duration after Ajmaline (ms) (mean +/- SD)** | 222.4±29.2 | 218.1±34.6 | 0.640 |

Abbreviations: ms: milliseconds; SD: standard deviation.

**Supplemental Table 9.** Varsome rules of pathogenicity.

| **SCN5A Variant** | **Varsome predictions of pathogenicity.** |
| --- | --- |
| c.4850_4852delTCT | **PP5 Strong**: ClinVar classifies this variant as Pathogenic, rated 2 stars, with 5 submissions, 8 publications () and no conflicts.  Using strength Strong because of the evidence presented by ClinVar.  **PM1 Moderate**: Hot-spot of length 68 base-pairs has 9 non-VUS coding variants (9 pathogenic and 0 benign), pathogenicity = 100.0%, qualifies as hot-spot.  **PM2 Moderate**: GnomAD exomes homozygous allele count = 0 is less than 3 threshold for recessive gene SCN5A (good gnomAD exomes coverage = 85.3). Variant not found in gnomAD genomes (good gnomAD genomes coverage = 26.8).  **PP3 Supporting**: Pathogenic computational verdict based on 1 pathogenic prediction from GERP vs no benign predictions.  **BP3 Supporting**: In frame variant in a repeat region of protein SCN5A_HUMAN of 4,720 base-pairs (source UniProt), and there are no known pathogenic variants within the repeat region. |
| c.809_812dup | **PVS1 Very Strong**: Null variant (frame-shift) affecting gene SCN5A, which is a known mechanism of disease (gene has 535 known pathogenic variants which is greater than minimum of 3), associated with Atrial fibrillation, familial 10, Long QT syndrome 3, Idiopathic ventricular fibrillation, Heart block, progressive, type IA, Heart block, nonprogressive, Sick sinus syndrome 1, autosomal recessive, Cardiomyopathy, dilated, 1E, Brugada syndrome 1 and Ventricular fibrillation, familial 1.  **PM2 Moderate**: Variant not found in gnomAD exomes (good gnomAD exomes coverage = 90.5). Variant not found in gnomAD genomes (good gnomAD genomes coverage = 34.8).  **PP3 Supporting**: Pathogenic computational verdict based on 1 pathogenic prediction from GERP vs no benign predictions. |
| c.1111C>T | **PVS1 Very Strong**: Null variant (nonsense) affecting gene SCN5A, which is a known mechanism of disease (gene has 535 known pathogenic variants which is greater than minimum of 3), associated with Atrial fibrillation, familial 10, Long QT syndrome 3, Idiopathic ventricular fibrillation, Heart block, progressive, type IA, Heart block, nonprogressive, Sick sinus syndrome 1, autosomal recessive, Cardiomyopathy, dilated, 1E, Brugada syndrome 1 and Ventricular fibrillation, familial 1.  **PM2 Moderate**: Variant not found in gnomAD exomes (good gnomAD exomes coverage = 51.4). Variant not found in gnomAD genomes (good gnomAD genomes coverage = 33.8).  **PP3 Supporting**: Pathogenic computational verdict based on 5 pathogenic predictions from BayesDel_addAF, DANN, EIGEN, FATHMM-MKL and MutationTaster vs no benign predictions. |
| c.3946C>T | **PVS1 Very Strong**: Null variant (nonsense) affecting gene SCN5A, which is a known mechanism of disease (gene has 535 known pathogenic variants which is greater than minimum of 3), associated with Atrial fibrillation, familial 10, Long QT syndrome 3, Idiopathic ventricular fibrillation, Heart block, progressive, type IA, Heart block, nonprogressive, Sick sinus syndrome 1, autosomal recessive, Cardiomyopathy, dilated, 1E, Brugada syndrome 1 and Ventricular fibrillation, familial 1.  **PP5 Strong**: ClinVar classifies this variant as Pathogenic, rated 2 stars, with 3 submissions, 2 publications () and no conflicts. Using strength Strong because of the evidence presented by ClinVar.  **PM2 Moderate**: Variant not found in gnomAD exomes (good gnomAD exomes coverage = 40.8). Variant not found in gnomAD genomes (good gnomAD genomes coverage = 32.8).  **PP3 Supporting**: Pathogenic computational verdict based on 4 pathogenic predictions from BayesDel_addAF, DANN, FATHMM-MKL and MutationTaster vs 1 benign prediction from EIGEN. |
| c.2091G>A | **PVS1 Very Strong**: Null variant (nonsense) affecting gene SCN5A, which is a known mechanism of disease (gene has 535 known pathogenic variants which is greater than minimum of 3), associated with Atrial fibrillation, familial 10, Long QT syndrome 3, Idiopathic ventricular fibrillation, Heart block, progressive, type IA, Heart block, nonprogressive, Sick sinus syndrome 1, autosomal recessive, Cardiomyopathy, dilated, 1E, Brugada syndrome 1 and Ventricular fibrillation, familial 1.  **PM2 Moderate**: Variant not found in gnomAD exomes (good gnomAD exomes coverage = 72.2). Variant not found in gnomAD genomes (good gnomAD genomes coverage = 34.4).  **PP5 Moderate**: VarSome users have linked 1 article stating the variant is Pathogenic (31590245). Using strength Moderate because of the evidence presented by VarSome users.  **PP3 Supporting**: Pathogenic computational verdict based on 5 pathogenic predictions from BayesDel_addAF, DANN, EIGEN, FATHMM-MKL and MutationTaster vs no benign predictions. |
| c.4437+5G>A | **PM2 Moderate**: Variant not found in gnomAD genomes (good gnomAD genomes coverage = 33.0).  **BP4 Supporting**: Benign computational verdict based on 1 benign prediction from DANN vs no pathogenic predictions and the position is not conserved (GERP++ rejected substitutions = 4.33 is less than 5.5). |
| c.4867C>T | **PVS1 Very Strong**: Null variant (nonsense) affecting gene SCN5A, which is a known mechanism of disease (gene has 535 known pathogenic variants which is greater than minimum of 3), associated with Atrial fibrillation, familial 10, Long QT syndrome 3, Idiopathic ventricular fibrillation, Heart block, progressive, type IA, Heart block, nonprogressive, Sick sinus syndrome 1, autosomal recessive, Cardiomyopathy, dilated, 1E, Brugada syndrome 1 and Ventricular fibrillation, familial 1.  **PP5 Strong**: ClinVar classifies this variant as Pathogenic, rated 2 stars, with 8 submissions, 9 publications () and no conflicts. Using strength Strong because of the evidence presented by ClinVar.  **PM2 Moderate**: GnomAD exomes homozygous allele count = 0 is less than 3 threshold for recessive gene SCN5A (good gnomAD exomes coverage = 92.8). Variant not found in gnomAD genomes (good gnomAD genomes coverage = 29.7).  **PP3 Supporting**: Pathogenic computational verdict based on 5 pathogenic predictions from BayesDel_addAF, DANN, EIGEN, FATHMM-MKL and MutationTaster vs no benign predictions. |
| c.4700_4701del | **PVS1 Very Strong**: Null variant (frame-shift) affecting gene SCN5A, which is a known mechanism of disease (gene has 535 known pathogenic variants which is greater than minimum of 3), associated with Atrial fibrillation, familial 10, Long QT syndrome 3, Idiopathic ventricular fibrillation, Heart block, progressive, type IA, Heart block, nonprogressive, Sick sinus syndrome 1, autosomal recessive, Cardiomyopathy, dilated, 1E, Brugada syndrome 1 and Ventricular fibrillation, familial 1.  **PM2 Moderate**: Variant not found in gnomAD exomes (good gnomAD exomes coverage = 87.7). Variant not found in gnomAD genomes (good gnomAD genomes coverage = 32.0).  **PP3 Supporting**: Pathogenic computational verdict based on 1 pathogenic prediction from GERP vs no benign predictions. |
| c.5830C>T | **PVS1 Very Strong**: Null variant (nonsense) affecting gene SCN5A, which is a known mechanism of disease (gene has 535 known pathogenic variants which is greater than minimum of 3), associated with Atrial fibrillation, familial 10, Long QT syndrome 3, Idiopathic ventricular fibrillation, Heart block, progressive, type IA, Heart block, nonprogressive, Sick sinus syndrome 1, autosomal recessive, Cardiomyopathy, dilated, 1E, Brugada syndrome 1 and Ventricular fibrillation, familial 1.  **PM2 Moderate**: GnomAD exomes homozygous allele count = 0 is less than 3 threshold for recessive gene SCN5A (good gnomAD exomes coverage = 54.7). GnomAD genomes homozygous allele count = 0 is less than 3 threshold for recessive gene SCN5A (good gnomAD genomes coverage = 33.0).  **PP3 Supporting**: Pathogenic computational verdict based on 5 pathogenic predictions from BayesDel_addAF, DANN, EIGEN, FATHMM-MKL and MutationTaster vs no benign predictions. |
| c.481G>A | **PM1 Moderate**: UniProt protein SCN5A_HUMAN trans-membrane region 'Helical' has 5 non-VUS, non-synonymous, coding variants (2 pathogenic and 3 benign), pathogenicity = 40.0% which is more than threshold 33.3%.  **PM2 Moderate**: GnomAD exomes homozygous allele count = 0 is less than 3 threshold for recessive gene SCN5A (good gnomAD exomes coverage = 41.1). Variant not found in gnomAD genomes (good gnomAD genomes coverage = 33.0).  **PP2 Supporting**: 366 out of 442 non-VUS missense variants in gene SCN5A are pathogenic = 82.8% which is more than threshold of 51.0%, and 535 out of 2,665 clinically reported variants in gene SCN5A are pathogenic = 20.1% which is more than threshold of 12.0%.  **PP3 Supporting**: Pathogenic computational verdict based on 12 pathogenic predictions from BayesDel_addAF, DANN, DEOGEN2, EIGEN, FATHMM-MKL, M-CAP, MVP, MutationAssessor, MutationTaster, PrimateAI, REVEL and SIFT vs no benign predictions.  **PP5 Supporting**: UniProt classifies this variant as Pathogenic, associated with Brugada syndrome, Brugada syndrome 1Progressive familial heart block 1A, related publications: 12106943, 19251209 and 20129283. |
| c.4867C>T | **PVS1 Very Strong**: Null variant (nonsense) affecting gene SCN5A, which is a known mechanism of disease (gene has 535 known pathogenic variants which is greater than minimum of 3), associated with Atrial fibrillation, familial 10, Long QT syndrome 3, Idiopathic ventricular fibrillation, Heart block, progressive, type IA, Heart block, nonprogressive, Sick sinus syndrome 1, autosomal recessive, Cardiomyopathy, dilated, 1E, Brugada syndrome 1 and Ventricular fibrillation, familial 1.  **PP5 Strong**: ClinVar classifies this variant as Pathogenic, rated 2 stars, with 8 submissions, 9 publications () and no conflicts. Using strength Strong because of the evidence presented by ClinVar.  **PM2 Moderate**: GnomAD exomes homozygous allele count = 0 is less than 3 threshold for recessive gene SCN5A (good gnomAD exomes coverage = 92.8). Variant not found in gnomAD genomes (good gnomAD genomes coverage = 29.7).  **PP3 Supporting**: Pathogenic computational verdict based on 5 pathogenic predictions from BayesDel_addAF, DANN, EIGEN, FATHMM-MKL and MutationTaster vs no benign predictions. |
| c.4294delA | **PVS1 Very Strong**: Null variant (frame-shift) affecting gene SCN5A, which is a known mechanism of disease (gene has 535 known pathogenic variants which is greater than minimum of 3), associated with Atrial fibrillation, familial 10, Long QT syndrome 3, Idiopathic ventricular fibrillation, Heart block, progressive, type IA, Heart block, nonprogressive, Sick sinus syndrome 1, autosomal recessive, Cardiomyopathy, dilated, 1E, Brugada syndrome 1 and Ventricular fibrillation, familial 1.  **PM2 Moderate**: Variant not found in gnomAD exomes (good gnomAD exomes coverage = 46.9). Variant not found in gnomAD genomes (good gnomAD genomes coverage = 33.1). |
| c.1140+2T>C | **PVS1 Very Strong**: Null variant (intronic within ±2 of splice site) affecting gene SCN5A, which is a known mechanism of disease (gene has 535 known pathogenic variants which is greater than minimum of 3), associated with Atrial fibrillation, familial 10, Long QT syndrome 3, Idiopathic ventricular fibrillation, Heart block, progressive, type IA, Heart block, nonprogressive, Sick sinus syndrome 1, autosomal recessive, Cardiomyopathy, dilated, 1E, Brugada syndrome 1 and Ventricular fibrillation, familial 1.  **PM2 Moderate**: Variant not found in gnomAD exomes (good gnomAD exomes coverage = 40.6). Variant not found in gnomAD genomes (good gnomAD genomes coverage = 33.6).  **PP3 Supporting**: Pathogenic computational verdict based on 5 pathogenic predictions from BayesDel_addAF, DANN, EIGEN, FATHMM-MKL and MutationTaster vs no benign predictions. |
| c.1890G>A | **PVS1 Strong**: Using strength Strong because Null variant (last base before splice site) affecting gene SCN5A, which is a known mechanism of disease (gene has 535 known pathogenic variants which is greater than minimum of 3), associated with Atrial fibrillation, familial 10, Long QT syndrome 3, Idiopathic ventricular fibrillation, Heart block, progressive, type IA, Heart block, nonprogressive, Sick sinus syndrome 1, autosomal recessive, Cardiomyopathy, dilated, 1E, Brugada syndrome 1 and Ventricular fibrillation, familial 1.  **PM2 Moderate**: GnomAD exomes homozygous allele count = 0 is less than 3 threshold for recessive gene SCN5A (good gnomAD exomes coverage = 24.8). Variant not found in gnomAD genomes (good gnomAD genomes coverage = 31.4).  **BP4 Supporting**: Benign computational verdict based on 1 benign prediction from DANN vs no pathogenic predictions and the position is not conserved (GERP++ rejected substitutions = 4.08 is less than 5.5). |
| c.1921delC | **PVS1 Very Strong**: Null variant (frame-shift) affecting gene SCN5A, which is a known mechanism of disease (gene has 535 known pathogenic variants which is greater than minimum of 3), associated with Atrial fibrillation, familial 10, Long QT syndrome 3, Idiopathic ventricular fibrillation, Heart block, progressive, type IA, Heart block, nonprogressive, Sick sinus syndrome 1, autosomal recessive, Cardiomyopathy, dilated, 1E, Brugada syndrome 1 and Ventricular fibrillation, familial 1.  **PM2 Moderate**: Variant not found in gnomAD exomes (good gnomAD exomes coverage = 38.7). Variant not found in gnomAD genomes (good gnomAD genomes coverage = 31.3).  **PP3 Supporting**: Pathogenic computational verdict based on 1 pathogenic prediction from GERP vs no benign predictions. |
| c.3840+1G>A | **PVS1 Very Strong**: Null variant (intronic within ±2 of splice site) affecting gene SCN5A, which is a known mechanism of disease (gene has 535 known pathogenic variants which is greater than minimum of 3), associated with Atrial fibrillation, familial 10, Long QT syndrome 3, Idiopathic ventricular fibrillation, Heart block, progressive, type IA, Heart block, nonprogressive, Sick sinus syndrome 1, autosomal recessive, Cardiomyopathy, dilated, 1E, Brugada syndrome 1 and Ventricular fibrillation, familial 1.  **PM2 Moderate**: GnomAD exomes homozygous allele count = 0 is less than 3 threshold for recessive gene SCN5A (good gnomAD exomes coverage = 55.7). Variant not found in gnomAD genomes (good gnomAD genomes coverage = 33.1).  **PP5 Moderate**: ClinVar classifies this variant as Pathogenic, rated 2 stars, with 2 submissions, 7 publications () and no conflicts. Using strength Moderate because of the evidence presented by ClinVar.  **PP3 Supporting**: Pathogenic computational verdict based on 5 pathogenic predictions from BayesDel_addAF, DANN, EIGEN, FATHMM-MKL and MutationTaster vs no benign predictions. |
| c.535C>T | **PVS1 Very Strong**: Null variant (nonsense) affecting gene SCN5A, which is a known mechanism of disease (gene has 535 known pathogenic variants which is greater than minimum of 3), associated with Atrial fibrillation, familial 10, Long QT syndrome 3, Idiopathic ventricular fibrillation, Heart block, progressive, type IA, Heart block, nonprogressive, Sick sinus syndrome 1, autosomal recessive, Cardiomyopathy, dilated, 1E, Brugada syndrome 1 and Ventricular fibrillation, familial 1.  **PP5 Strong**: ClinVar classifies this variant as Pathogenic, rated 2 stars, with 3 submissions, 4 publications () and no conflicts. Using strength Strong because of the evidence presented by ClinVar.  **PM2 Moderate**: GnomAD exomes homozygous allele count = 0 is less than 3 threshold for recessive gene SCN5A (good gnomAD exomes coverage = 58.6). Variant not found in gnomAD genomes (good gnomAD genomes coverage = 33.6).  **PP3 Supporting**: Pathogenic computational verdict based on 5 pathogenic predictions from BayesDel_addAF, DANN, EIGEN, FATHMM-MKL and MutationTaster vs no benign predictions. |
| c.2893C>T | **PM2 Moderate**: GnomAD exomes homozygous allele count = 0 is less than 3 threshold for recessive gene SCN5A (good gnomAD exomes coverage = 47.5). Variant not found in gnomAD genomes (good gnomAD genomes coverage = 30.8).  **PP2 Supporting**: 366 out of 442 non-VUS missense variants in gene SCN5A are pathogenic = 82.8% which is more than threshold of 51.0%, and 535 out of 2,665 clinically reported variants in gene SCN5A are pathogenic = 20.1% which is more than threshold of 12.0%.  **PP3 Supporting**: Pathogenic computational verdict based on 11 pathogenic predictions from BayesDel_addAF, DANN, DEOGEN2, EIGEN, FATHMM-MKL, M-CAP, MVP, MutationAssessor, MutationTaster, REVEL and SIFT vs 1 benign prediction from PrimateAI.  **PP5 Supporting**: UniProt classifies this variant as Pathogenic, associated with Brugada syndrome 1, related publications: 11901046, 19272188 and 20129283. |
| c.481G>A | **PM1 Moderate**: UniProt protein SCN5A_HUMAN trans-membrane region 'Helical' has 5 non-VUS, non-synonymous, coding variants (2 pathogenic and 3 benign), pathogenicity = 40.0% which is more than threshold 33.3%.  **PM2 Moderate**: GnomAD exomes homozygous allele count = 0 is less than 3 threshold for recessive gene SCN5A (good gnomAD exomes coverage = 41.1). Variant not found in gnomAD genomes (good gnomAD genomes coverage = 33.0).  **PP2 Supporting**: 366 out of 442 non-VUS missense variants in gene SCN5A are pathogenic = 82.8% which is more than threshold of 51.0%, and 535 out of 2,665 clinically reported variants in gene SCN5A are pathogenic = 20.1% which is more than threshold of 12.0%.  **PP3 Supporting**: Pathogenic computational verdict based on 12 pathogenic predictions from BayesDel_addAF, DANN, DEOGEN2, EIGEN, FATHMM-MKL, M-CAP, MVP, MutationAssessor, MutationTaster, PrimateAI, REVEL and SIFT vs no benign predictions.  **PP5 Supporting**: UniProt classifies this variant as Pathogenic, associated with Brugada syndrome, Brugada syndrome 1Progressive familial heart block 1A, related publications: 12106943, 19251209 and 20129283. |
| c.2314G>A | **PM2 Moderate**: GnomAD exomes homozygous allele count = 0 is less than 3 threshold for recessive gene SCN5A (good gnomAD exomes coverage = 66.7). Variant not found in gnomAD genomes (good gnomAD genomes coverage = 32.3).  **PP2 Supporting**: 366 out of 442 non-VUS missense variants in gene SCN5A are pathogenic = 82.8% which is more than threshold of 51.0%, and 535 out of 2,665 clinically reported variants in gene SCN5A are pathogenic = 20.1% which is more than threshold of 12.0%.  **PP3 Supporting**: Pathogenic computational verdict based on 11 pathogenic predictions from BayesDel_addAF, DANN, DEOGEN2, EIGEN, FATHMM-MKL, M-CAP, MutationAssessor, MutationTaster, PrimateAI, REVEL and SIFT vs 1 benign prediction from MVP.  **PP5 Supporting**: UniProt classifies this variant as Pathogenic, associated with Brugada syndrome 1, Congenital long QT syndromeLong QT syndrome 3, related publications: 19716085 and 20129283.  **BS3 Strong**: A VarSome user has reported this variant is classified Benign in article 32533946 and that it is confirmed by a functional study. |
| c.4813G>T | **PVS1 Strong**: Using strength Strong because Null variant (last base before splice site) affecting gene SCN5A, which is a known mechanism of disease (gene has 535 known pathogenic variants which is greater than minimum of 3), associated with Atrial fibrillation, familial 10, Long QT syndrome 3, Idiopathic ventricular fibrillation, Heart block, progressive, type IA, Heart block, nonprogressive, Sick sinus syndrome 1, autosomal recessive, Cardiomyopathy, dilated, 1E, Brugada syndrome 1 and Ventricular fibrillation, familial 1.  **PM1 Moderate**: UniProt protein SCN5A_HUMAN trans-membrane region 'Helical' has 4 non-VUS, non-synonymous, coding variants (4 pathogenic and 0 benign), pathogenicity = 100.0% which is more than threshold 33.3%.  **PM2 Moderate**: GnomAD exomes homozygous allele count = 0 is less than 3 threshold for recessive gene SCN5A (good gnomAD exomes coverage = 44.0). Variant not found in gnomAD genomes (good gnomAD genomes coverage = 32.3).  **PM5 Moderate**: Alternative variant chr3:38595770 C⇒G (Gly1605His) is classified Pathogenic, 1 star, by ClinVar (and confirmed using ACMG).  **PP2 Supporting**: 366 out of 442 non-VUS missense variants in gene SCN5A are pathogenic = 82.8% which is more than threshold of 51.0%, and 535 out of 2,665 clinically reported variants in gene SCN5A are pathogenic = 20.1% which is more than threshold of 12.0%.  **PP3 Supporting**: Pathogenic computational verdict based on 12 pathogenic predictions from BayesDel_addAF, DANN, DEOGEN2, EIGEN, FATHMM-MKL, M-CAP, MVP, MutationAssessor, MutationTaster, PrimateAI, REVEL and SIFT vs no benign predictions. |
| c.4534C>T | **PM2 Moderate**: GnomAD exomes homozygous allele count = 0 is less than 3 threshold for recessive gene SCN5A (good gnomAD exomes coverage = 55.6). Variant not found in gnomAD genomes (good gnomAD genomes coverage = 30.4).  **PP2 Supporting**: 366 out of 442 non-VUS missense variants in gene SCN5A are pathogenic = 82.8% which is more than threshold of 51.0%, and 535 out of 2,665 clinically reported variants in gene SCN5A are pathogenic = 20.1% which is more than threshold of 12.0%.  **PP3 Supporting**: Pathogenic computational verdict based on 11 pathogenic predictions from BayesDel_addAF, DANN, DEOGEN2, FATHMM-MKL, M-CAP, MVP, MutationAssessor, MutationTaster, PrimateAI, REVEL and SIFT vs 1 benign prediction from EIGEN.  **PP5 Supporting**: UniProt classifies this variant as Pathogenic, associated with Brugada syndrome 1Brugada syndrome 1, related publications: 10690282, 12106943, 19251209, 20129283 and 22158541. |
| c.4057G>A | **PM1 Moderate**: UniProt protein SCN5A_HUMAN trans-membrane region 'Helical' has 8 non-VUS, non-synonymous, coding variants (7 pathogenic and 1 benign), pathogenicity = 87.5% which is more than threshold 33.3%.  **PM2 Moderate**: GnomAD exomes homozygous allele count = 0 is less than 3 threshold for recessive gene SCN5A (good gnomAD exomes coverage = 76.2). Variant not found in gnomAD genomes (good gnomAD genomes coverage = 33.8).  **PP2 Supporting**: 366 out of 442 non-VUS missense variants in gene SCN5A are pathogenic = 82.8% which is more than threshold of 51.0%, and 535 out of 2,665 clinically reported variants in gene SCN5A are pathogenic = 20.1% which is more than threshold of 12.0%.  **PP3 Supporting**: Pathogenic computational verdict based on 12 pathogenic predictions from BayesDel_addAF, DANN, DEOGEN2, EIGEN, FATHMM-MKL, M-CAP, MVP, MutationAssessor, MutationTaster, PrimateAI, REVEL and SIFT vs no benign predictions.  **BS3 Strong**: A VarSome user has reported this variant is classified Benign in article 32533946 and that it is confirmed by a functional study. |
| c.4312C>T | **PM2 Moderate**: Variant not found in gnomAD exomes (good gnomAD exomes coverage = 37.3). Variant not found in gnomAD genomes (good gnomAD genomes coverage = 31.5).  **PP2 Supporting**: 366 out of 442 non-VUS missense variants in gene SCN5A are pathogenic = 82.8% which is more than threshold of 51.0%, and 535 out of 2,665 clinically reported variants in gene SCN5A are pathogenic = 20.1% which is more than threshold of 12.0%.  **PP3 Supporting**: Pathogenic computational verdict based on 11 pathogenic predictions from BayesDel_addAF, DANN, DEOGEN2, EIGEN, FATHMM-MKL, M-CAP, MVP, MutationAssessor, MutationTaster, REVEL and SIFT vs 1 benign prediction from PrimateAI.  **PP5 Supporting**: ClinVar classifies this variant as Likely Pathogenic, rated 1 star, criteria provided, single submitter, with 1 submission. |
| c.692C>T | **PM1 Moderate**: Hot-spot of length 61 base-pairs has 9 non-VUS coding variants (9 pathogenic and 0 benign), pathogenicity = 100.0%, qualifies as hot-spot.  **PM2 Moderate**: Variant not found in gnomAD exomes (good gnomAD exomes coverage = 42.0). Variant not found in gnomAD genomes (good gnomAD genomes coverage = 34.7).  **PP2 Supporting**: 366 out of 442 non-VUS missense variants in gene SCN5A are pathogenic = 82.8% which is more than threshold of 51.0%, and 535 out of 2,665 clinically reported variants in gene SCN5A are pathogenic = 20.1% which is more than threshold of 12.0%.  **PP3 Supporting**: Pathogenic computational verdict based on 11 pathogenic predictions from BayesDel_addAF, DANN, DEOGEN2, EIGEN, FATHMM-MKL, M-CAP, MVP, MutationAssessor, MutationTaster, REVEL and SIFT vs no benign predictions. |
| c.5000T>A | **PM1 Moderate**: UniProt protein SCN5A_HUMAN trans-membrane region 'Helical' has 4 non-VUS, non-synonymous, coding variants (4 pathogenic and 0 benign), pathogenicity = 100.0% which is more than threshold 33.3%.  **PM2 Moderate**: Variant not found in gnomAD exomes (good gnomAD exomes coverage = 99.6). Variant not found in gnomAD genomes (good gnomAD genomes coverage = 31.9).  **PM5 Moderate**: Alternative variant chr3:38592864 C⇒T (Val1667Ile) is classified Pathogenic by UniProt Variants (and confirmed using ACMG).  **PP2 Supporting**: 366 out of 442 non-VUS missense variants in gene SCN5A are pathogenic = 82.8% which is more than threshold of 51.0%, and 535 out of 2,665 clinically reported variants in gene SCN5A are pathogenic = 20.1% which is more than threshold of 12.0%.  **PP3 Supporting**: Pathogenic computational verdict based on 11 pathogenic predictions from BayesDel_addAF, DEOGEN2, EIGEN, FATHMM-MKL, M-CAP, MVP, MutationAssessor, MutationTaster, PrimateAI, REVEL and SIFT vs no benign predictions (1 uncertain prediction from DANN). |
| c.86_87delinsTG | **PM2 Moderate**: Variant not found in gnomAD exomes (good gnomAD exomes coverage = 63.9). Variant not found in gnomAD genomes (good gnomAD genomes coverage = 30.9).  **PP2 Supporting**: 366 out of 442 non-VUS missense variants in gene SCN5A are pathogenic = 82.8% which is more than threshold of 51.0%, and 535 out of 2,665 clinically reported variants in gene SCN5A are pathogenic = 20.1% which is more than threshold of 12.0%.  **BP4 Supporting**: Benign computational verdict based on 1 benign prediction from GERP vs no pathogenic predictions. |
| c.4501C>G | **PM1 Moderate**: Hot-spot of length 61 base-pairs has 13 non-VUS coding variants (13 pathogenic and 0 benign), pathogenicity = 100.0%, qualifies as hot-spot.  **PM2 Moderate**: GnomAD exomes homozygous allele count = 0 is less than 3 threshold for recessive gene SCN5A (good gnomAD exomes coverage = 67.4). Variant not found in gnomAD genomes (good gnomAD genomes coverage = 29.9).  **PP2 Supporting**: 366 out of 442 non-VUS missense variants in gene SCN5A are pathogenic = 82.8% which is more than threshold of 51.0%, and 535 out of 2,665 clinically reported variants in gene SCN5A are pathogenic = 20.1% which is more than threshold of 12.0%.  **PP3 Supporting**: Pathogenic computational verdict based on 11 pathogenic predictions from BayesDel_addAF, DANN, DEOGEN2, FATHMM-MKL, M-CAP, MVP, MutationAssessor, MutationTaster, PrimateAI, REVEL and SIFT vs 1 benign prediction from EIGEN.  **PP5 Supporting**: UniProt classifies this variant as Pathogenic, associated with Brugada syndrome 1, Congenital long QT syndromeLong QT syndrome 3, related publications: 10973849, 19716085 and 20129283. |
| c.1144C>T | **PM1 Moderate**: UniProt protein SCN5A_HUMAN intra-membrane domain 'Pore-forming' has 11 non-VUS, non-synonymous, coding variants (11 pathogenic and 0 benign), pathogenicity = 100.0% which is more than threshold 33.3%.  **PM2 Moderate**: Variant not found in gnomAD exomes (good gnomAD exomes coverage = 48.3). Variant not found in gnomAD genomes (good gnomAD genomes coverage = 32.3).  **PP2 Supporting**: 366 out of 442 non-VUS missense variants in gene SCN5A are pathogenic = 82.8% which is more than threshold of 51.0%, and 535 out of 2,665 clinically reported variants in gene SCN5A are pathogenic = 20.1% which is more than threshold of 12.0%.  **PP3 Supporting**: Pathogenic computational verdict based on 11 pathogenic predictions from BayesDel_addAF, DANN, DEOGEN2, EIGEN, FATHMM-MKL, M-CAP, MutationAssessor, MutationTaster, PrimateAI, REVEL and SIFT vs 1 benign prediction from MVP. |
| c.2441G>A | **PS3 Strong**: A VarSome user has reported this variant is classified Pathogenic in article 32533946 and that it is confirmed by a functional study.  **PM1 Moderate**: UniProt protein SCN5A_HUMAN trans-membrane region 'Helical' has 5 non-VUS, non-synonymous, coding variants (5 pathogenic and 0 benign), pathogenicity = 100.0% which is more than threshold 33.3%.  **PM2 Moderate**: GnomAD exomes homozygous allele count = 0 is less than 3 threshold for recessive gene SCN5A (good gnomAD exomes coverage = 46.8). GnomAD genomes homozygous allele count = 0 is less than 3 threshold for recessive gene SCN5A (good gnomAD genomes coverage = 30.1).  **PM5 Moderate**: Alternative variant chr3:38627529 G⇒A (Arg814Trp) is classified Pathogenic, 2 stars, by ClinVar (and confirmed using ACMG).  **PP2 Supporting**: 366 out of 442 non-VUS missense variants in gene SCN5A are pathogenic = 82.8% which is more than threshold of 51.0%, and 535 out of 2,665 clinically reported variants in gene SCN5A are pathogenic = 20.1% which is more than threshold of 12.0%.  **PP3 Supporting**: Pathogenic computational verdict based on 12 pathogenic predictions from BayesDel_addAF, DANN, DEOGEN2, EIGEN, FATHMM-MKL, M-CAP, MVP, MutationAssessor, MutationTaster, PrimateAI, REVEL and SIFT vs no benign predictions.  **PP5 Supporting**: UniProt classifies this variant as Pathogenic, associated with Brugada syndrome 1. |
| c.5089T>C | **PM1 Moderate**: UniProt protein SCN5A_HUMAN intra-membrane domain 'Pore-forming' has 8 non-VUS, non-synonymous, coding variants (8 pathogenic and 0 benign), pathogenicity = 100.0% which is more than threshold 33.3%.  **PM2 Moderate**: Variant not found in gnomAD exomes (good gnomAD exomes coverage = 99.7). Variant not found in gnomAD genomes (good gnomAD genomes coverage = 30.8).  **PP2 Supporting**: 366 out of 442 non-VUS missense variants in gene SCN5A are pathogenic = 82.8% which is more than threshold of 51.0%, and 535 out of 2,665 clinically reported variants in gene SCN5A are pathogenic = 20.1% which is more than threshold of 12.0%.  **PP3 Supporting**: Pathogenic computational verdict based on 12 pathogenic predictions from BayesDel_addAF, DANN, DEOGEN2, EIGEN, FATHMM-MKL, M-CAP, MVP, MutationAssessor, MutationTaster, PrimateAI, REVEL and SIFT vs no benign predictions. |
| c.3214_3215delinsTC | **PM2 Moderate**: Variant not found in gnomAD exomes (good gnomAD exomes coverage = 43.5). Variant not found in gnomAD genomes (good gnomAD genomes coverage = 32.5).  **PP2 Supporting**: 366 out of 442 non-VUS missense variants in gene SCN5A are pathogenic = 82.8% which is more than threshold of 51.0%, and 535 out of 2,665 clinically reported variants in gene SCN5A are pathogenic = 20.1% which is more than threshold of 12.0%.  **PP3 Supporting**: Pathogenic computational verdict based on 1 pathogenic prediction from GERP vs no benign predictions. |
| c.1045G>A | **PS3 Strong**: A VarSome user has reported this variant is classified Pathogenic in article 32533946 and that it is confirmed by a functional study.  **PM1 Moderate**: Hot-spot of length 61 base-pairs has 7 non-VUS coding variants (6 pathogenic and 1 benign), pathogenicity = 85.7%, qualifies as hot-spot.  **PM2 Moderate**: GnomAD exomes homozygous allele count = 0 is less than 3 threshold for recessive gene SCN5A (good gnomAD exomes coverage = 58.2). GnomAD genomes homozygous allele count = 0 is less than 3 threshold for recessive gene SCN5A (good gnomAD genomes coverage = 33.4).  **PP2 Supporting**: 366 out of 442 non-VUS missense variants in gene SCN5A are pathogenic = 82.8% which is more than threshold of 51.0%, and 535 out of 2,665 clinically reported variants in gene SCN5A are pathogenic = 20.1% which is more than threshold of 12.0%.  **BP4 Supporting**: Benign computational verdict based on 8 benign predictions from BayesDel_addAF, DANN, EIGEN, MVP, MutationAssessor, PrimateAI, REVEL and SIFT vs 4 pathogenic predictions from DEOGEN2, FATHMM-MKL, M-CAP and MutationTaster and the position is not conserved (GERP++ rejected substitutions = 4.73 is less than 5.5). |
| c.3673G>A | **PS3 Strong**: A VarSome user has reported this variant is classified Pathogenic in article 32533946 and that it is confirmed by a functional study.  **PM2 Moderate**: GnomAD exomes homozygous allele count = 0 is less than 3 threshold for recessive gene SCN5A (good gnomAD exomes coverage = 69.4). Variant not found in gnomAD genomes (good gnomAD genomes coverage = 33.0).  **PP2 Supporting**: 366 out of 442 non-VUS missense variants in gene SCN5A are pathogenic = 82.8% which is more than threshold of 51.0%, and 535 out of 2,665 clinically reported variants in gene SCN5A are pathogenic = 20.1% which is more than threshold of 12.0%.  **PP3 Supporting**: Pathogenic computational verdict based on 12 pathogenic predictions from BayesDel_addAF, DANN, DEOGEN2, EIGEN, FATHMM-MKL, M-CAP, MVP, MutationAssessor, MutationTaster, PrimateAI, REVEL and SIFT vs no benign predictions.  **PP5 Supporting**: UniProt classifies this variant as Pathogenic, associated with Brugada syndrome 1, Congenital long QT syndromeLong QT syndrome 3, related publications: 12106943, 15840476 and 20129283. |
| c.2542A>G | **PM1 Moderate**: Hot-spot of length 61 base-pairs has 7 non-VUS coding variants (7 pathogenic and 0 benign), pathogenicity = 100.0%, qualifies as hot-spot.  **PM2 Moderate**: Variant not found in gnomAD exomes (good gnomAD exomes coverage = 94.3). Variant not found in gnomAD genomes (good gnomAD genomes coverage = 31.7).  **PP2 Supporting**: 366 out of 442 non-VUS missense variants in gene SCN5A are pathogenic = 82.8% which is more than threshold of 51.0%, and 535 out of 2,665 clinically reported variants in gene SCN5A are pathogenic = 20.1% which is more than threshold of 12.0%.  **PP3 Supporting**: Pathogenic computational verdict based on 11 pathogenic predictions from BayesDel_addAF, DANN, DEOGEN2, EIGEN, FATHMM-MKL, M-CAP, MutationAssessor, MutationTaster, PrimateAI, REVEL and SIFT vs no benign predictions (1 uncertain prediction from MVP). |
| c.733C>A | **PM2 Moderate**: GnomAD exomes homozygous allele count = 0 is less than 3 threshold for recessive gene SCN5A (good gnomAD exomes coverage = 80.2). Variant not found in gnomAD genomes (good gnomAD genomes coverage = 33.5).  **PP2 Supporting**: 366 out of 442 non-VUS missense variants in gene SCN5A are pathogenic = 82.8% which is more than threshold of 51.0%, and 535 out of 2,665 clinically reported variants in gene SCN5A are pathogenic = 20.1% which is more than threshold of 12.0%.  **PP3 Supporting**: Pathogenic computational verdict based on 11 pathogenic predictions from BayesDel_addAF, DANN, DEOGEN2, EIGEN, FATHMM-MKL, M-CAP, MVP, MutationTaster, PrimateAI, REVEL and SIFT vs 1 benign prediction from MutationAssessor.  **PP5 Supporting**: UniProt classifies this variant as Pathogenic, associated with Congenital long QT syndromeLong QT syndrome 3, related publications: 15840476. |
| c.880G>A | **PM1 Moderate**: UniProt protein SCN5A_HUMAN disulfide bond domain 'disulfide bond_280-335' has 16 non-VUS, non-synonymous, coding variants (9 pathogenic and 7 benign), pathogenicity = 56.2% which is more than threshold 33.3%.  **PM2 Moderate**: GnomAD exomes homozygous allele count = 0 is less than 3 threshold for recessive gene SCN5A (good gnomAD exomes coverage = 65.3). GnomAD genomes homozygous allele count = 0 is less than 3 threshold for recessive gene SCN5A (good gnomAD genomes coverage = 34.9).  **PP2 Supporting**: 366 out of 442 non-VUS missense variants in gene SCN5A are pathogenic = 82.8% which is more than threshold of 51.0%, and 535 out of 2,665 clinically reported variants in gene SCN5A are pathogenic = 20.1% which is more than threshold of 12.0%.  **PP5 Supporting**: UniProt classifies this variant as Pathogenic, associated with Atrial fibrillation, familial, 10, Brugada syndrome, Brugada syndrome 1, Brugada syndrome 1, Dilated cardiomyopathy 1E, Long QT syndrome 3, Paroxysmal familial ventricular fibrillation 1, Progressive familial heart block type 1A, SUDDEN INFANT DEATH SYNDROMESick sinus syndrome 1, autosomal recessive, related publications: 11901046.  **BP4 Supporting**: Benign computational verdict based on 7 benign predictions from EIGEN, FATHMM-MKL, MutationAssessor, MutationTaster, PrimateAI, REVEL and SIFT vs 5 pathogenic predictions from BayesDel_addAF, DANN, DEOGEN2, M-CAP and MVP and the position is not conserved (GERP++ rejected substitutions = 4.47 is less than 5.5). |
| c.5227G>A | **PP5 Very Strong**: ClinVar classifies this variant as Pathogenic, rated 2 stars, with 3 submissions, 6 publications () and no conflicts.  UniProt classifies this variant as Pathogenic, associated with Brugada syndromeBrugada syndrome 1, related publications: 15023552, 20129283 and 23420830. Using strength Very Strong because of the evidence presented by ClinVar and UniProt.  **PS1 Strong**: Equivalent variant chr3:38592636 C⇒G (Gly1743Arg) is classified Pathogenic by UniProt Variants (and confirmed using ACMG).  **PM1 Moderate**: Hot-spot of length 61 base-pairs has 8 non-VUS coding variants (7 pathogenic and 1 benign), pathogenicity = 87.5%, qualifies as hot-spot.  **PM2 Moderate**: Variant not found in gnomAD exomes (good gnomAD exomes coverage = 91.3). Variant not found in gnomAD genomes (good gnomAD genomes coverage = 28.8).  **PM5 Moderate**: Alternative variant chr3:38592635 C⇒T (Gly1743Glu) is classified Pathogenic by UniProt Variants (and confirmed using ACMG).  **PP2 Supporting**: 366 out of 442 non-VUS missense variants in gene SCN5A are pathogenic = 82.8% which is more than threshold of 51.0%, and 535 out of 2,665 clinically reported variants in gene SCN5A are pathogenic = 20.1% which is more than threshold of 12.0%.  **PP3 Supporting**: Pathogenic computational verdict based on 11 pathogenic predictions from BayesDel_addAF, DANN, DEOGEN2, EIGEN, FATHMM-MKL, M-CAP, MVP, MutationAssessor, MutationTaster, REVEL and SIFT vs 1 benign prediction from PrimateAI. |
| c.4720G>A | **PS3 Strong**: A VarSome user has reported this variant is classified Pathogenic in article 32533946 and that it is confirmed by a functional study.  **PM1 Moderate**: Hot-spot of length 61 base-pairs has 6 non-VUS coding variants (5 pathogenic and 1 benign), pathogenicity = 83.3%, qualifies as hot-spot.  **PM2 Moderate**: Variant not found in gnomAD exomes (good gnomAD exomes coverage = 83.5). Variant not found in gnomAD genomes (good gnomAD genomes coverage = 30.8).  **PP2 Supporting**: 366 out of 442 non-VUS missense variants in gene SCN5A are pathogenic = 82.8% which is more than threshold of 51.0%, and 535 out of 2,665 clinically reported variants in gene SCN5A are pathogenic = 20.1% which is more than threshold of 12.0%.  **PP3 Supporting**: Pathogenic computational verdict based on 11 pathogenic predictions from BayesDel_addAF, DANN, DEOGEN2, EIGEN, FATHMM-MKL, M-CAP, MVP, MutationAssessor, MutationTaster, REVEL and SIFT vs 1 benign prediction from PrimateAI. |
| c.655C>T | **PM1 Moderate**: Hot-spot of length 61 base-pairs has 9 non-VUS coding variants (9 pathogenic and 0 benign), pathogenicity = 100.0%, qualifies as hot-spot.  **PM2 Moderate**: GnomAD exomes homozygous allele count = 0 is less than 3 threshold for recessive gene SCN5A (good gnomAD exomes coverage = 40.4). Variant not found in gnomAD genomes (good gnomAD genomes coverage = 32.7).  **PP2 Supporting**: 366 out of 442 non-VUS missense variants in gene SCN5A are pathogenic = 82.8% which is more than threshold of 51.0%, and 535 out of 2,665 clinically reported variants in gene SCN5A are pathogenic = 20.1% which is more than threshold of 12.0%.  **PP3 Supporting**: Pathogenic computational verdict based on 11 pathogenic predictions from BayesDel_addAF, DANN, DEOGEN2, EIGEN, FATHMM-MKL, M-CAP, MVP, MutationAssessor, MutationTaster, REVEL and SIFT vs no benign predictions. |
| c.4140C>G | **PS1 Strong**: Equivalent variant chr3:38601743 G⇒T (Asn1380Lys) is classified Pathogenic by a VarSome user in article 32533946 (and confirmed using ACMG).  **PM1 Moderate**: Hot-spot of length 61 base-pairs has 7 non-VUS coding variants (7 pathogenic and 0 benign), pathogenicity = 100.0%, qualifies as hot-spot.  **PM2 Moderate**: Variant not found in gnomAD exomes (good gnomAD exomes coverage = 74.9). Variant not found in gnomAD genomes (good gnomAD genomes coverage = 34.2).  **PP2 Supporting**: 366 out of 442 non-VUS missense variants in gene SCN5A are pathogenic = 82.8% which is more than threshold of 51.0%, and 535 out of 2,665 clinically reported variants in gene SCN5A are pathogenic = 20.1% which is more than threshold of 12.0%.  **PP3 Supporting**: Pathogenic computational verdict based on 11 pathogenic predictions from BayesDel_addAF, DANN, DEOGEN2, EIGEN, FATHMM-MKL, M-CAP, MVP, MutationAssessor, MutationTaster, REVEL and SIFT vs 1 benign prediction from PrimateAI. |
| c.2989G>A | **PM1 Moderate**: Hot-spot of length 61 base-pairs has 5 non-VUS coding variants (5 pathogenic and 0 benign), pathogenicity = 100.0%, qualifies as hot-spot.  **PM2 Moderate**: GnomAD exomes homozygous allele count = 0 is less than 3 threshold for recessive gene SCN5A (good gnomAD exomes coverage = 39.8). GnomAD genomes homozygous allele count = 0 is less than 3 threshold for recessive gene SCN5A (good gnomAD genomes coverage = 30.3).  **PM5 Moderate**: Alternative variant chr3:38622660 GC⇒CT (Ala997Ser) is classified Pathogenic by UniProt Variants (and confirmed using ACMG). Alternative variant chr3:38622661 C⇒A (Ala997Ser) is classified Pathogenic by UniProt Variants (and confirmed using ACMG).  **PP2 Supporting**: 366 out of 442 non-VUS missense variants in gene SCN5A are pathogenic = 82.8% which is more than threshold of 51.0%, and 535 out of 2,665 clinically reported variants in gene SCN5A are pathogenic = 20.1% which is more than threshold of 12.0%.  **BP4 Supporting**: Benign computational verdict based on 10 benign predictions from BayesDel_addAF, DANN, DEOGEN2, EIGEN, FATHMM-MKL, MutationAssessor, MutationTaster, PrimateAI, REVEL and SIFT vs 2 pathogenic predictions from M-CAP and MVP and the position is not conserved (GERP++ rejected substitutions = -10.8 is less than 5.5). |
| c.2414C>T | **PM1 Moderate**: UniProt protein SCN5A_HUMAN trans-membrane region 'Helical' has 5 non-VUS, non-synonymous, coding variants (5 pathogenic and 0 benign), pathogenicity = 100.0% which is more than threshold 33.3%.  **PM2 Moderate**: GnomAD exomes homozygous allele count = 0 is less than 3 threshold for recessive gene SCN5A (good gnomAD exomes coverage = 37.8). GnomAD genomes homozygous allele count = 0 is less than 3 threshold for recessive gene SCN5A (good gnomAD genomes coverage = 33.1).  **PP2 Supporting**: 366 out of 442 non-VUS missense variants in gene SCN5A are pathogenic = 82.8% which is more than threshold of 51.0%, and 535 out of 2,665 clinically reported variants in gene SCN5A are pathogenic = 20.1% which is more than threshold of 12.0%.  **PP3 Supporting**: Pathogenic computational verdict based on 12 pathogenic predictions from BayesDel_addAF, DANN, DEOGEN2, EIGEN, FATHMM-MKL, M-CAP, MVP, MutationAssessor, MutationTaster, PrimateAI, REVEL and SIFT vs no benign predictions. |
| c.2039G>A | **BP6 Strong**: ClinVar classifies this variant as Likely Benign, rated 2 stars, with 3 submissions, 4 publications () and no conflicts. Using strength Strong because of the evidence presented by ClinVar.  **BP4 Supporting**: Benign computational verdict based on 8 benign predictions from DANN, EIGEN, FATHMM-MKL, MutationAssessor, MutationTaster, PrimateAI, REVEL and SIFT vs 4 pathogenic predictions from BayesDel_addAF, DEOGEN2, M-CAP and MVP and the position is not conserved (GERP++ rejected substitutions = 2.02 is less than 5.5).  **PM2 Moderate**: Variant not found in gnomAD exomes (good gnomAD exomes coverage = 53.3).  GnomAD genomes homozygous allele count = 0 is less than 3 threshold for recessive gene SCN5A (good gnomAD genomes coverage = 32.7).  **PP2 Supporting**: 366 out of 442 non-VUS missense variants in gene SCN5A are pathogenic = 82.8% which is more than threshold of 51.0%, and 535 out of 2,665 clinically reported variants in gene SCN5A are pathogenic = 20.1% which is more than threshold of 12.0%.  **PP5 Supporting**: UniProt classifies this variant as Pathogenic, associated with Long QT syndrome 3SUDDEN INFANT DEATH SYNDROME. |
| c.481G>A | **PM1 Moderate**: UniProt protein SCN5A_HUMAN trans-membrane region 'Helical' has 5 non-VUS, non-synonymous, coding variants (2 pathogenic and 3 benign), pathogenicity = 40.0% which is more than threshold 33.3%.  **PM2 Moderate**: GnomAD exomes homozygous allele count = 0 is less than 3 threshold for recessive gene SCN5A (good gnomAD exomes coverage = 41.1). Variant not found in gnomAD genomes (good gnomAD genomes coverage = 33.0).  **PP2 Supporting**: 366 out of 442 non-VUS missense variants in gene SCN5A are pathogenic = 82.8% which is more than threshold of 51.0%, and 535 out of 2,665 clinically reported variants in gene SCN5A are pathogenic = 20.1% which is more than threshold of 12.0%.  **PP3 Supporting**: Pathogenic computational verdict based on 12 pathogenic predictions from BayesDel_addAF, DANN, DEOGEN2, EIGEN, FATHMM-MKL, M-CAP, MVP, MutationAssessor, MutationTaster, PrimateAI, REVEL and SIFT vs no benign predictions.  **PP5 Supporting**: UniProt classifies this variant as Pathogenic, associated with Brugada syndrome, Brugada syndrome 1Progressive familial heart block 1A, related publications: 12106943, 19251209 and 20129283. |
| c.1041C>A | **PM1 Moderate**: Hot-spot of length 61 base-pairs has 7 non-VUS coding variants (6 pathogenic and 1 benign), pathogenicity = 85.7%, qualifies as hot-spot.  **PM2 Moderate**: Variant not found in gnomAD exomes (good gnomAD exomes coverage = 58.4). Variant not found in gnomAD genomes (good gnomAD genomes coverage = 33.5).  **PP2 Supporting**: 366 out of 442 non-VUS missense variants in gene SCN5A are pathogenic = 82.8% which is more than threshold of 51.0%, and 535 out of 2,665 clinically reported variants in gene SCN5A are pathogenic = 20.1% which is more than threshold of 12.0%.  **PP3 Supporting**: Pathogenic computational verdict based on 11 pathogenic predictions from BayesDel_addAF, DANN, DEOGEN2, EIGEN, FATHMM-MKL, M-CAP, MVP, MutationAssessor, MutationTaster, REVEL and SIFT vs 1 benign prediction from PrimateAI. |
| c.5102T>G | **PM1 Moderate**: Hot-spot of length 61 base-pairs has 8 non-VUS coding variants (8 pathogenic and 0 benign), pathogenicity = 100.0%, qualifies as hot-spot.  **PM2 Moderate**: Variant not found in gnomAD exomes (good gnomAD exomes coverage = 99.6). Variant not found in gnomAD genomes (good gnomAD genomes coverage = 30.8).  **PP2 Supporting**: 366 out of 442 non-VUS missense variants in gene SCN5A are pathogenic = 82.8% which is more than threshold of 51.0%, and 535 out of 2,665 clinically reported variants in gene SCN5A are pathogenic = 20.1% which is more than threshold of 12.0%.  **PP3 Supporting**: Pathogenic computational verdict based on 12 pathogenic predictions from BayesDel_addAF, DANN, DEOGEN2, EIGEN, FATHMM-MKL, M-CAP, MVP, MutationAssessor, MutationTaster, PrimateAI, REVEL and SIFT vs no benign predictions. |
| c.6004G>A | **PM2 Moderate**: GnomAD exomes homozygous allele count = 0 is less than 3 threshold for recessive gene SCN5A (good gnomAD exomes coverage = 54.9). Variant not found in gnomAD genomes (good gnomAD genomes coverage = 32.8).  **PP2 Supporting**: 366 out of 442 non-VUS missense variants in gene SCN5A are pathogenic = 82.8% which is more than threshold of 51.0%, and 535 out of 2,665 clinically reported variants in gene SCN5A are pathogenic = 20.1% which is more than threshold of 12.0%.  **BP4 Supporting**: Benign computational verdict based on 9 benign predictions from BayesDel_addAF, DEOGEN2, EIGEN, FATHMM-MKL, MVP, MutationAssessor, MutationTaster, PrimateAI and REVEL vs 3 pathogenic predictions from DANN, M-CAP and SIFT and the position is not conserved (GERP++ rejected substitutions = 4.6 is less than 5.5). |
| c.5129C>T | **PP5 Very Strong**: ClinVar classifies this variant as Pathogenic, rated 2 stars, with 9 submissions, 10 publications () and no conflicts.  UniProt classifies this variant as Pathogenic, associated with Brugada syndrome 1Familial paroxysmal ventricular fibrillation 1, related publications: 10940383. Using strength Very Strong because of the evidence presented by ClinVar and UniProt.  **PM1 Moderate**: Hot-spot of length 61 base-pairs has 8 non-VUS coding variants (8 pathogenic and 0 benign), pathogenicity = 100.0%, qualifies as hot-spot.  **PM2 Moderate**: GnomAD exomes homozygous allele count = 0 is less than 3 threshold for recessive gene SCN5A (good gnomAD exomes coverage = 98.9). Variant not found in gnomAD genomes (good gnomAD genomes coverage = 29.9).  **PP2 Supporting**: 366 out of 442 non-VUS missense variants in gene SCN5A are pathogenic = 82.8% which is more than threshold of 51.0%, and 535 out of 2,665 clinically reported variants in gene SCN5A are pathogenic = 20.1% which is more than threshold of 12.0%.  **PP3 Supporting**: Pathogenic computational verdict based on 12 pathogenic predictions from BayesDel_addAF, DANN, DEOGEN2, EIGEN, FATHMM-MKL, M-CAP, MVP, MutationAssessor, MutationTaster, PrimateAI, REVEL and SIFT vs no benign predictions. |
| c.589G>T | **PM1 Moderate**: Hot-spot of length 61 base-pairs has 5 non-VUS coding variants (5 pathogenic and 0 benign), pathogenicity = 100.0%, qualifies as hot-spot.  **PM2 Moderate**: Variant not found in gnomAD exomes (good gnomAD exomes coverage = 52.9). Variant not found in gnomAD genomes (good gnomAD genomes coverage = 34.1).  **PM5 Moderate**: Alternative variant chr3:38662355 T⇒C (Asp197Gly) is classified Likely Pathogenic, 1 star, by ClinVar (and confirmed using ACMG). Alternative variant chr3:38662356 C⇒G (Asp197His) is classified Likely Pathogenic, 1 star, by ClinVar (and confirmed using ACMG).  **PP2 Supporting**: 366 out of 442 non-VUS missense variants in gene SCN5A are pathogenic = 82.8% which is more than threshold of 51.0%, and 535 out of 2,665 clinically reported variants in gene SCN5A are pathogenic = 20.1% which is more than threshold of 12.0%.  **PP3 Supporting**: Pathogenic computational verdict based on 11 pathogenic predictions from BayesDel_addAF, DANN, DEOGEN2, EIGEN, FATHMM-MKL, M-CAP, MVP, MutationAssessor, MutationTaster, REVEL and SIFT vs 1 benign prediction from PrimateAI. |
| c.3917G>A | **PM1 Moderate**: Hot-spot of length 61 base-pairs has 7 non-VUS coding variants (7 pathogenic and 0 benign), pathogenicity = 100.0%, qualifies as hot-spot.  **PM2 Moderate**: GnomAD exomes homozygous allele count = 0 is less than 3 threshold for recessive gene SCN5A (good gnomAD exomes coverage = 49.5). Variant not found in gnomAD genomes (good gnomAD genomes coverage = 32.1).  **PP2 Supporting**: 366 out of 442 non-VUS missense variants in gene SCN5A are pathogenic = 82.8% which is more than threshold of 51.0%, and 535 out of 2,665 clinically reported variants in gene SCN5A are pathogenic = 20.1% which is more than threshold of 12.0%.  **PP3 Supporting**: Pathogenic computational verdict based on 12 pathogenic predictions from BayesDel_addAF, DANN, DEOGEN2, EIGEN, FATHMM-MKL, M-CAP, MVP, MutationAssessor, MutationTaster, PrimateAI, REVEL and SIFT vs no benign predictions. |
| c.422T>A | **PM1 Moderate**: Hot-spot of length 61 base-pairs has 6 non-VUS coding variants (5 pathogenic and 1 benign), pathogenicity = 83.3%, qualifies as hot-spot.  **PM2 Moderate**: GnomAD exomes homozygous allele count = 0 is less than 3 threshold for recessive gene SCN5A (good gnomAD exomes coverage = 51.1). GnomAD genomes homozygous allele count = 0 is less than 3 threshold for recessive gene SCN5A (good gnomAD genomes coverage = 34.3).  **PP2 Supporting**: 366 out of 442 non-VUS missense variants in gene SCN5A are pathogenic = 82.8% which is more than threshold of 51.0%, and 535 out of 2,665 clinically reported variants in gene SCN5A are pathogenic = 20.1% which is more than threshold of 12.0%.  **PP3 Supporting**: Pathogenic computational verdict based on 12 pathogenic predictions from BayesDel_addAF, DANN, DEOGEN2, EIGEN, FATHMM-MKL, M-CAP, MVP, MutationAssessor, MutationTaster, PrimateAI, REVEL and SIFT vs no benign predictions. |
| c.4894C>T | **PM1 Moderate**: Hot-spot of length 61 base-pairs has 7 non-VUS coding variants (7 pathogenic and 0 benign), pathogenicity = 100.0%, qualifies as hot-spot.  **PM2 Moderate**: GnomAD exomes homozygous allele count = 0 is less than 3 threshold for recessive gene SCN5A (good gnomAD exomes coverage = 96.4). Variant not found in gnomAD genomes (good gnomAD genomes coverage = 30.7).  **PP5 Moderate**: ClinVar classifies this variant as Pathogenic, rated 2 stars, with 2 submissions, 2 publications () and no conflicts. Using strength Moderate because of the evidence presented by ClinVar.  **PP2 Supporting**: 366 out of 442 non-VUS missense variants in gene SCN5A are pathogenic = 82.8% which is more than threshold of 51.0%, and 535 out of 2,665 clinically reported variants in gene SCN5A are pathogenic = 20.1% which is more than threshold of 12.0%.  **PP3 Supporting**: Pathogenic computational verdict based on 12 pathogenic predictions from BayesDel_addAF, DANN, DEOGEN2, EIGEN, FATHMM-MKL, M-CAP, MVP, MutationAssessor, MutationTaster, PrimateAI, REVEL and SIFT vs no benign predictions. |
| c.2182G>A | **PM1 Moderate**: UniProt protein SCN5A_HUMAN trans-membrane region 'Helical' has 4 non-VUS, non-synonymous, coding variants (4 pathogenic and 0 benign), pathogenicity = 100.0% which is more than threshold 33.3%.  **PM2 Moderate**: GnomAD exomes homozygous allele count = 0 is less than 3 threshold for recessive gene SCN5A (good gnomAD exomes coverage = 75.3). GnomAD genomes homozygous allele count = 0 is less than 3 threshold for recessive gene SCN5A (good gnomAD genomes coverage = 33.5).  **PP2 Supporting**: 366 out of 442 non-VUS missense variants in gene SCN5A are pathogenic = 82.8% which is more than threshold of 51.0%, and 535 out of 2,665 clinically reported variants in gene SCN5A are pathogenic = 20.1% which is more than threshold of 12.0%.  **PP3 Supporting**: Pathogenic computational verdict based on 8 pathogenic predictions from BayesDel_addAF, DANN, DEOGEN2, FATHMM-MKL, M-CAP, MutationTaster, REVEL and SIFT vs 4 benign predictions from EIGEN, MVP, MutationAssessor and PrimateAI. |
| c.1144C>T | **PM1 Moderate**: UniProt protein SCN5A_HUMAN intra-membrane domain 'Pore-forming' has 11 non-VUS, non-synonymous, coding variants (11 pathogenic and 0 benign), pathogenicity = 100.0% which is more than threshold 33.3%.  **PM2 Moderate**: Variant not found in gnomAD exomes (good gnomAD exomes coverage = 48.3). Variant not found in gnomAD genomes (good gnomAD genomes coverage = 32.3).  **PP2 Supporting**: 366 out of 442 non-VUS missense variants in gene SCN5A are pathogenic = 82.8% which is more than threshold of 51.0%, and 535 out of 2,665 clinically reported variants in gene SCN5A are pathogenic = 20.1% which is more than threshold of 12.0%.  **PP3 Supporting**: Pathogenic computational verdict based on 11 pathogenic predictions from BayesDel_addAF, DANN, DEOGEN2, EIGEN, FATHMM-MKL, M-CAP, MutationAssessor, MutationTaster, PrimateAI, REVEL and SIFT vs 1 benign prediction from MVP. |
| c.4516C>A | **PM1 Moderate**: Hot-spot of length 61 base-pairs has 7 non-VUS coding variants (7 pathogenic and 0 benign), pathogenicity = 100.0%, qualifies as hot-spot.  **PM2 Moderate**: Variant not found in gnomAD exomes (good gnomAD exomes coverage = 63.1). Variant not found in gnomAD genomes (good gnomAD genomes coverage = 30.1).  **PP2 Supporting**: 366 out of 442 non-VUS missense variants in gene SCN5A are pathogenic = 82.8% which is more than threshold of 51.0%, and 535 out of 2,665 clinically reported variants in gene SCN5A are pathogenic = 20.1% which is more than threshold of 12.0%.  **PP3 Supporting**: Pathogenic computational verdict based on 12 pathogenic predictions from BayesDel_addAF, DANN, DEOGEN2, EIGEN, FATHMM-MKL, M-CAP, MVP, MutationAssessor, MutationTaster, PrimateAI, REVEL and SIFT vs no benign predictions. |
| c.5863A>T | **PM2 Moderate**: GnomAD exomes homozygous allele count = 0 is less than 3 threshold for recessive gene SCN5A (good gnomAD exomes coverage = 46.4). Variant not found in gnomAD genomes (good gnomAD genomes coverage = 32.2).  **PP2 Supporting**: 366 out of 442 non-VUS missense variants in gene SCN5A are pathogenic = 82.8% which is more than threshold of 51.0%, and 535 out of 2,665 clinically reported variants in gene SCN5A are pathogenic = 20.1% which is more than threshold of 12.0%.  **PP3 Supporting**: Pathogenic computational verdict based on 8 pathogenic predictions from BayesDel_addAF, DANN, DEOGEN2, FATHMM-MKL, M-CAP, MutationAssessor, MutationTaster and SIFT vs 4 benign predictions from EIGEN, MVP, PrimateAI and REVEL. |

**Supplemental Figure 1.** *SCN5A* carrier with drug-induced type 1 ECG pattern (ECG on the left, top and bottom panels), who experienced previous spontaneous VT/VF episodes. Epicardial mapping demonstrated a large area (8.5 cm^2^) of abnormal potentials, which increased to 21 cm^2^ after ajmaline administration.

**
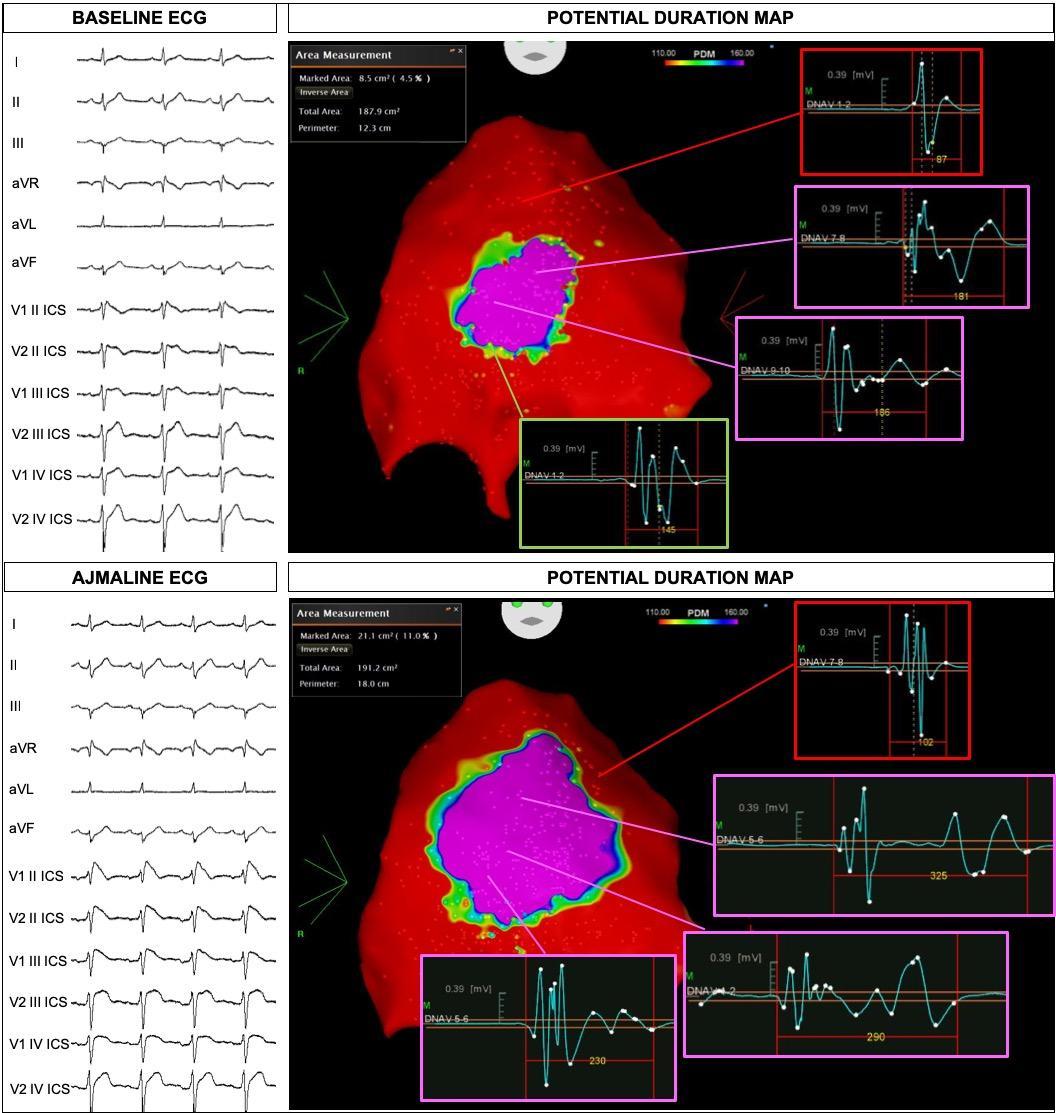
**

**Supplemental Figure 2.** Example of an *SCN5A* mutation carrier, with spontaneous type 1 ECG BrS pattern (left panel), who survived a previous CA. Epicardial mapping demonstrated a large area (18.5 cm^2^) of abnormal potentials (right panel).

**
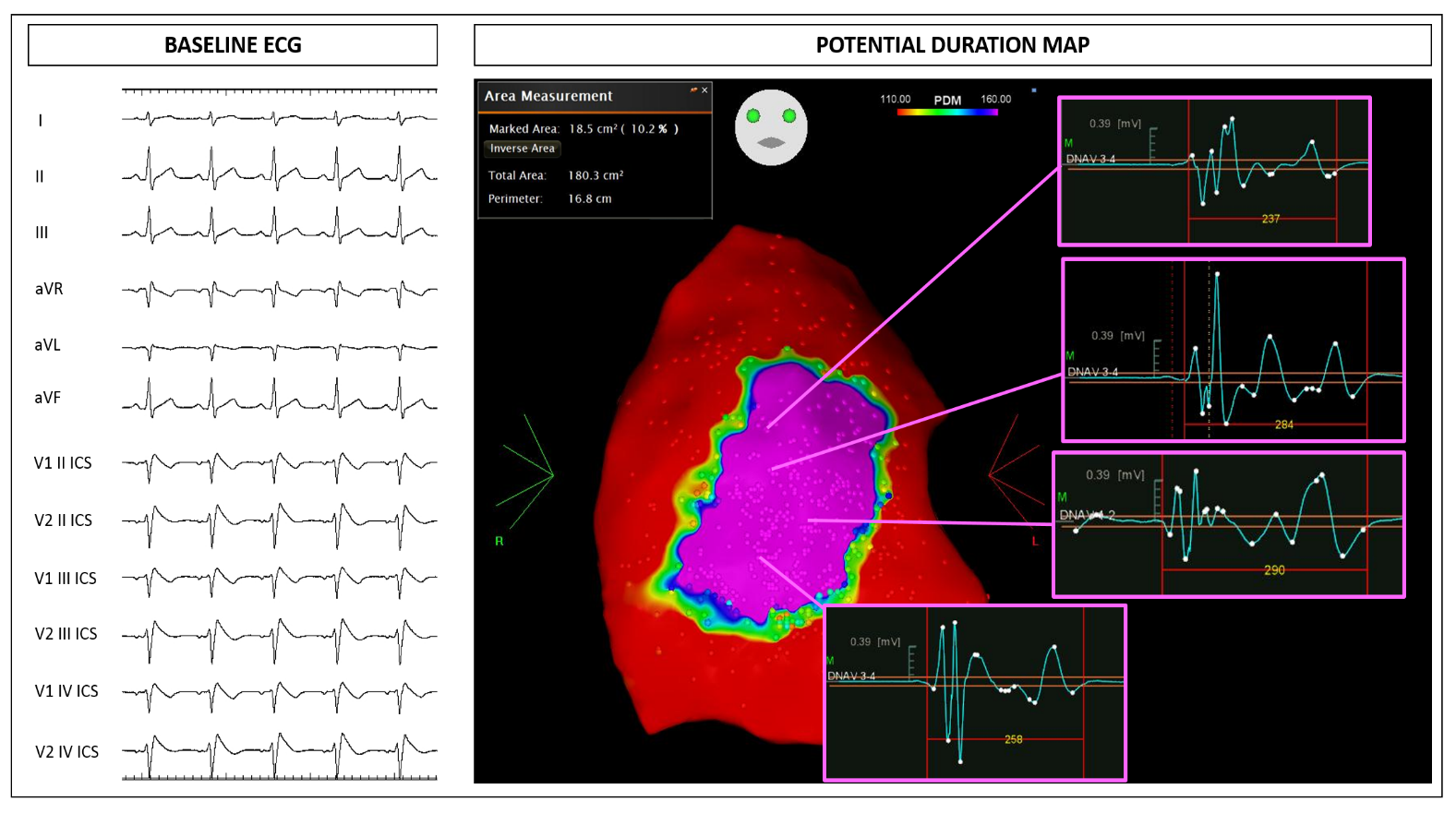
**

**Supplemental Figure 3.** *SCN5A* mutation carriers with spontaneous type 1 ECG pattern have larger substrate size (1.5-fold) than patients without spontaneous type 1 ECG pattern.


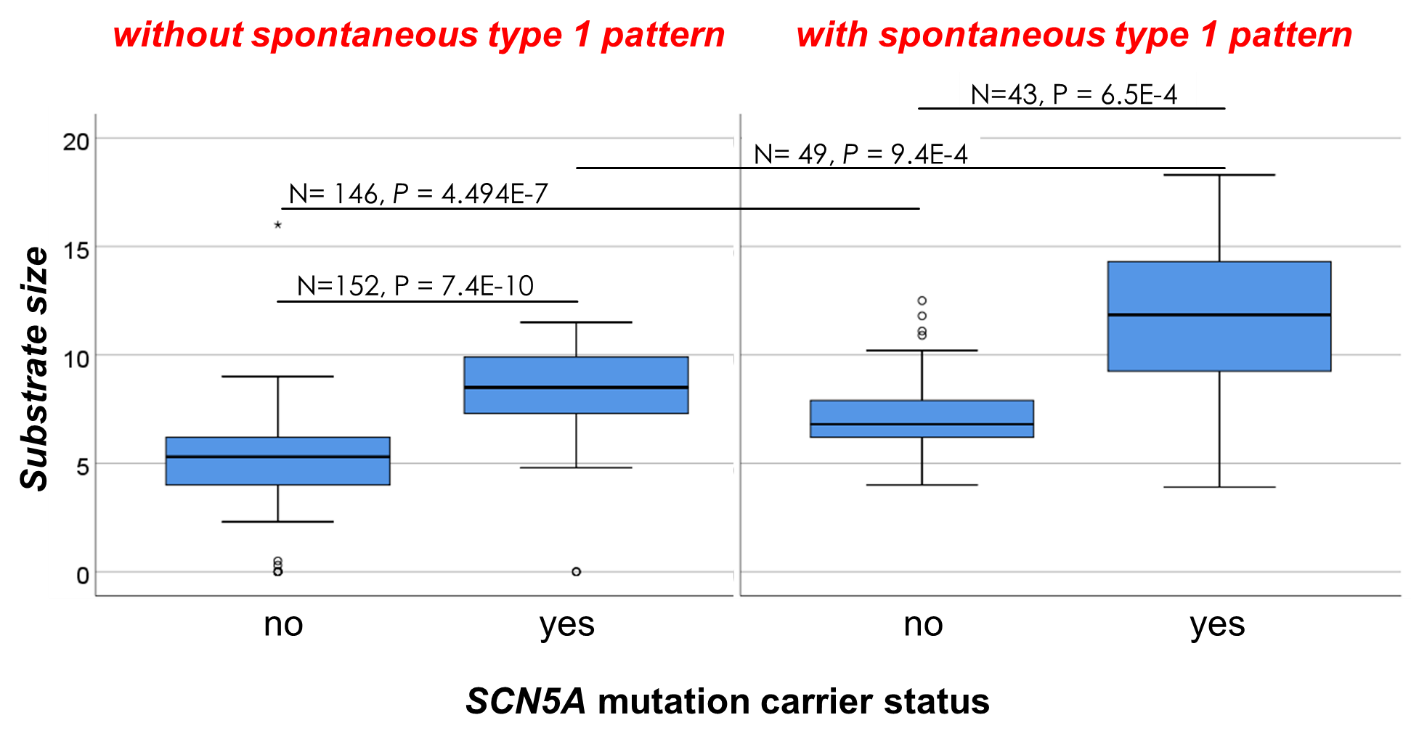

Supplement: ehaa942_Supplementary_Data [file ehaa942_supplementary_data.docx]
